# Late Sodium Current of the Heart: Where Do We Stand and Where Are We Going?

**DOI:** 10.3390/ph15020231

**Published:** 2022-02-15

**Authors:** Balázs Horváth, Norbert Szentandrássy, János Almássy, Csaba Dienes, Zsigmond Máté Kovács, Péter P. Nánási, Tamas Banyasz

**Affiliations:** 1 Department of Physiology, University of Debrecen, 4032 Debrecen, Hungary; horvath.balazs@med.unideb.hu (B.H.); szentandrassy.norbert@med.unideb.hu (N.S.); almassy.janos@med.unideb.hu (J.A.); dienes.csaba@med.unideb.hu (C.D.); kovacs.zsigmond@med.unideb.hu (Z.M.K.); nanasi.peter@med.unideb.hu (P.P.N.); 2 Department of Basic Medical Sciences, Faculty of Dentistry, University of Debrecen, 4032 Debrecen, Hungary; 3 Department of Dental Physiology and Pharmacology, University of Debrecen, 4032 Debrecen, Hungary

**Keywords:** late sodium current, cardiac sodium channel, arrhythmia

## Abstract

Late sodium current has long been linked to dysrhythmia and contractile malfunction in the heart. Despite the increasing body of accumulating information on the subject, our understanding of its role in normal or pathologic states is not complete. Even though the role of late sodium current in shaping action potential under physiologic circumstances is debated, it’s unquestioned role in arrhythmogenesis keeps it in the focus of research. Transgenic mouse models and isoform-specific pharmacological tools have proved useful in understanding the mechanism of late sodium current in health and disease. This review will outline the mechanism and function of cardiac late sodium current with special focus on the recent advances of the area.

## 1. Introduction

The late component of cardiac sodium current has been the subject of extensive research since firstly described. It is known to modulate the repolarization of the AP [1,2,3,4,5], calcium homeostasis [6,7,8] and mechanical activity [9,10,11,12]. Inherited mutations or pathologic conditions, including myocardial ischemia, pressure overload, and hypertrophy are reported to facilitate late sodium current, resulting in abnormal electric activity of the heart [13,14,15,16,17]. Additionally, specific inhibition of the current was shown to reduce the risk of arrhythmias [18,19,20,21,22]. Hence, late sodium current seemed a promising candidate in the therapy of cardiac arrhythmias and other heart diseases. The growing number of research papers and comprehensive reviews published in the last decade indicates the increasing interest in late sodium current [23,24,25,26,27,28].

One of the major obstacles in the late sodium current research is the tiny magnitude of the current. Accurate recording, especially in the presence of the huge transient phase, is a major challenge with the currently available experimental armory. Another limiting factor for progress is the limited availability of specific late sodium current inhibitors. To circumvent these difficulties, many researchers choose to use anemone toxin II (ATX-II) [29] or transgenic models [30] in order amplify the current magnitude. These models offer increased methodical reliability in electrophysiological measurements but do not necessarily mirror the physiologic conditions. Thus, when interpreting these data, particular caution must be exercised.

The late sodium current is not exclusive to cardiac cells. Its presence has been demonstrated in neurons [31,32], vascular smooth muscle [33], tumors [34], and pancreatic ß-cells [35]. Despite its minute extent, it seems to have a profound impact on the action potential morphology and calcium homeostasis in multiple cell types. In this review we outline our current understanding on the function of cardiac late sodium current in health and disease, with special emphasis on the last year’s development. The aim of this review is to report and discuss the major and most recent works that help to understand the precise function of this current in the heart.

## 2. A Brief History of Cardiac Late Sodium Current Research

Tracing back the history of cellular electrophysiology we can identify numerous observations pointing toward the existence of a late sodium current in the excitable membrane as early as the 1960s. In 1962 and 1963, Bernard Frankenhaeuser from the Karolinska Institute reported a *“small inward current”* after “*sufficiently long time for the sodium transport mechanism to be inactivated*” in the Xenopus Ranvier node. It is important to highlight that the “*sufficiently long time*” was between 4 and 6 milliseconds in these works. The observed current “*was so small it was barely detectable*.” Since the potassium ion as a potential charge carrier was excluded by further experiments and austere reasoning, “*it was concluded therefore that the membrane showed a secondary small increase of sodium permeability*” [36,37,38,39]. Possible physiologic roles or pathologic potential of the newly observed current was not considered in these publications. The concept and the name of late sodium current was introduced by Dubois and Bergman in 1975, reporting a persistent, tetrodotoxin (TTX) sensitive current from a frog Ranvier node. The current was interpreted as a minor subpopulation of voltage gated sodium channels (VGSC) that failed to inactivate [40]. Importantly, the current magnitude was measured 140 ms after the activation.

First reports indicating the presence of late sodium current in cardiac muscle soon followed these early observations. Coraboeuf et al. reported shortening of the canine Purkinje action potential (AP) following the application of a low concentration of TTX without reducing the amplitude of “*the normal rapid sodium current*” [41]. In the conclusions, authors proposed two critical features for cardiac late sodium current: (a) the presence of this small, persistent sodium current during the whole plateau of cardiac AP and (b) the contribution of non-cardiac voltage dependent sodium channels to cardiac AP. In the same year, Attwell et al. demonstrated that the voltage dependence of the TTX sensitive, non-inactivating sodium current overlaps with the “window region” of steady state inactivation (SSI) and activation curves in sheep Purkinje fibers [42]. The “window theory” for the mechanism behind late sodium current has been established with this observation. Importantly, Attwell et al. used very long (5–10 s) depolarizing pulses in these experiments. Further studies conducted in single channel models revealed that late sodium current is formed by two different activity patterns of VGSCs. Following the rapid, transient phase of sodium current caused by “normal” or “transient mode” opening, VGSCs can reopen, showing either “late scattered mode” or “burst mode” activity. According to these observations, the late activation was present in less than 4% of patches [43]. In addition to the “window theory”, a new mechanism believed to underlie late sodium current was suggested by Clancy et al. in 2003. The concept of “non-equilibrium gating” proposed that the changing voltage during the plateau accelerates the recovery from inactivation in VGSCs [44].

Late sodium current research was tremendously boosted when clinical observations and experimental studies linked this relatively small current to cardiac arrhythmias. Late sodium current was shown to be upregulated by pathologic conditions like hypoxia, free radicals, or ischemic metabolites [45,46,47]. Then, the upregulated plateau current can imbalance the fine equilibrium of ion currents shaping AP, leading to an increased propensity of electric disturbances. Furthermore, selective inhibition of late sodium current was shown to be clinically beneficial in pathologic models. Numerous studies demonstrated that inhibition of late sodium current can suppress arrhythmias, improve angina, and reduce contractile dysfunction [48,49,50,51,52,53]. Recent observations obtained by modern techniques indicate that early observations underestimated the magnitude of late sodium current. New measurements raised the possibility that late sodium current magnitude during the plateau is comparable with that of major potassium currents [1,54].

## 3. Cardiac Sodium Channels: Structure and Morphology

Although the primary goal of this paper is to discuss the recent development of research on the late sodium current, it is helpful to begin with a brief review on the general properties of cardiac sodium channels. VGSCs are made of a large pore forming an α subunit and one or two ß subunits. The α subunit alone, when properly positioned in the membrane, is able to respond to voltage changes and conduct Na^+^. The ß subunit, when co-assemble with the pore-forming main unit, modulates the gating and facilitates the gathering of channels at the intercalated disks [55].

### 3.1. Molecular Identity of Cardiac Sodium Channels

In mammals, nine members (Na_v_1.1–Na_v_1.9) of the family of voltage-activated fast sodium channels have been identified [56]. The classification is based on the pore forming (α) subunit, and the different subtypes display substantial differences in kinetic properties and drug sensitivity [57,58]. The dominant sodium channel isoform in the heart is Na_v_1.5, also known as *h1* or *skm II*. The channel is often referred to as SCN5A, after the encoding gene [56]. Beyond this dominant “*cardiac*” subtype, several “*non-cardiac*” isoforms were identified in the myocardium with immunochemistry, or RT-PCR technique. The following non-cardiac isoforms were detected in heart until now: Na_v_1.1, Na_v_1.2, Na_v_1.3, Na_v_1.4, Na_v_1.6, and Na_v_1.8 [59,60,61,62,63,64,65,66,67]. The expression level and the contribution of these isoforms to the total sodium current shows substantial variation among species. In mice, the cardiac isoform constitutes 70.3% of the total expressed VGSCs in the sarcolemma. In large mammals, like pigs and humans, the share of cardiac isoform is higher than 95%. Na_v_1.3 and Na_v_1.4 were the most abundant non-cardiac forms in all species studied [57,67].

The contribution of non-cardiac isoforms to late sodium current is not necessarily proportional to the expression level, especially under pathologic conditions. Biet et al. reported that while the contribution of non-cardiac VGSCs to the transient (peak) sodium current is between 5–10%, in the case of late sodium current the contribution is as high as 44% in dogs [61]. The difference can be explained by the different gating/kinetic properties of the isoforms. Similar observations were made by Yang et al. According to their data, the non-cardiac VGSCs are responsible for the 38% of the late sodium current in mice. They demonstrated the presence of non-cardiac VGSCs is rabbit ventricular cells too, but due to the unusually slow inactivation of the sodium current, the quantitative evaluation is not conclusive [62]. Yang et al. focused on the presence of neuronal isoform Na_v_1.8 in their study. This isoform differs significantly from Na_v_1.5 in the voltage dependence of activation and inactivation kinetics because both curves are shifted toward positive voltages [62]. This positive shift results in a similar shift of the window region making Na_v_1.8 an excellent candidate to generate a powerful plateau current. Interestingly, polymorphism of the Na_v_1.8 encoding gene *SCN10A* was linked to modulation of heart rate and myocardial conduction as well as arrhythmias [68,69,70]. Furthermore, the presence of *SCN10A* variants were associated with late sodium current in human myocardium [71,72,73,74], and Yang et al. demonstrated that the selective inhibition of Na_v_1.8 could reduce late sodium current [62]. These observations pointed to the direction of Na_v_1.8, and this non-cardiac sodium channel isoform could be a major component of late sodium current in the heart in both health and disease. Recently, Bengel et al. addressed this problem using a transgenic mouse model and specific Na_v_1.8 inhibitors [4]. In a set of elegant experiments, they convincingly demonstrated that Na_v_1.8 contributes significantly to shaping late sodium current and AP in mouse hearts. First, they showed that specific Na_v_1.8 inhibitors reduce ATX-II stimulated late sodium current in wild-type, but not in *SCN10A^-/-^* cardiomyocytes. The have also shown that Na_v_1.8 inhibitors shorten ATX-II lengthened AP in WT but not in *SCN10A^-/-^* cells.

These accumulating observations convincingly demonstrate that the Na_v_1.5 is unquestionably the dominant isoform in the heart, but non-cardiac VGSCs are also present. Numerous studies reported the presence of Na_v_1.8 from various regions of both healthy and diseased hearts. Most studies found well-detectable mRNA or protein levels [7,75,76], but there are reports on low expression levels too [75,76]. The contribution of Na_v_1.8 to late sodium current is well-established with both direct and indirect observations in the human heart. Na_v_1.8 specific inhibitors were shown to reduce late sodium current in various regions of the human myocardium [7,75,77,78], but Casini et al. reported the absence of Na_v_1.8 based late sodium current in the human left atrium [76]. In accordance with this, Na_v_1.8 inhibition was shown to shorten AP [79,80]. The presence of other non-cardiac VGSCs were also reported from human myocardium [75], but their role in the electrophysiology of human myocardium remains to be determined.

Depending on the species or experimental model, non-cardiac VGSCs may contribute to late sodium current in different extent. Consideration of species differences seems critically important when translating experimental data to human. For example, the presence of Na_v_1.8 has been demonstrated in human myocardium [77], therefore, the mouse model seems to be an advantageous choice to study late sodium current. In contrast, the translation of rabbit late sodium current data to human heart requires caution because rabbit heart lacks Na_v_1.8 [76]. Furthermore, the upregulation of late sodium current is reported from diverse pathologic conditions, and the increased depolarization drive is suspected to lead to arrhythmias. Experimental observations indicate that different VGSC isoforms upregulate in different degrees in various states. For instance, neuronal isoforms are reported to upregulate in a pressure-overload model [78]. In conclusion, we should see clearly that cardiac late sodium current is a composite current and the proportion of the isoforms may vary.

Six subtypes of ß subunits (ß1, ß1A, ß1B, ß2–ß4) encoded by four genes have been identified until now, but the presence of ß1A is not proven in mammals [81,82,83,84]. The different ß variants co-assemble with various affinities to different α subunits [85]. Na_v_1.5 is shown to combine with all four subtypes, but Na_v_1.4 associates only with ß1 [86]. The ß subunits display substantial morphological and functional differences. While ß2 and ß4 bind to the α-subunit via disulfide bond, the ß1 and ß3 associate non-covalently [81]. The ß-subunits are multifunctional proteins. They modulate the gating, voltage dependence, kinetics, expression, and trafficking of the VGSC α-subunit [87]. Co-expression of Na_v_1.5 with ß1B results in increased sodium current density in the heterologous expression system. Association of ß3 with Na_v_1.5 shifts the voltage dependence of inactivation and decreases the rate of inactivation in CHO cells. In the ventricular myocytes of ß1_,_ knockout mice transient and persistent sodium currents were found to be increased due to increased Na_v_1.5 expression [88]. Mutations within ß-subunits are shown to lead to clinical arrhythmias [85]. Recently, Angsutararux et al. published interesting data on ß-subunit mutants causing Brugada syndrome, or atrial fibrillation [89]. They demonstrated that mutations in the same positions of the ß1 and ß3 subunits led to the same disease phenotype despite the differential regulation they exert on the α subunit. Furthermore, they proposed that ß3 subunit exerts its effects via modulating the function of the voltage sensor domain in the pore forming subunit. At the same time, ß1 variants show more subtle effects via channel gating and modulating α subunit or ß1 expression. These data further support the insight that in spite of the molecular homology, various ß subunits exert diverse regulation on the pore forming subunit of VGSC.

The intracellular domain of ß4 subtype is postulated to play a central role in the mechanism of resurgent current in neurons [90]. The presence of resurgent current has not been proved in hearts, but the ß4 subunit has been identified in atrial and ventricular myocytes [85]. Therefore, resurgent current could possibly be present in cardiac muscle too.

Additionally, ß-subunits are known to be involved in multiple, non-VGSC related functions, like regulation of cell adhesion and migration.

### 3.2. Morphology of VGSCs

Sodium channels are made up of a larger pore-forming α-subunit and one or more smaller auxiliary ß-subunits. The α-subunit is a highly organized structure including four homologous domains between the C and N terminus (DI-DIV). Each domain is composed of six transmembrane segments (S1–S6). The pore is formed by S5–S6 segments with the intramembrane linker between them (P-loop) that functions as a selectivity filter. The S4 segments contain a high number of positively charged amino acids and serve as voltage sensors.

According to our current understanding, the α-subunits of the VGSCs can interact with each other during trafficking in the cytosol and are able to form functional dimers in the cell membrane. The dimerization has been demonstrated with Na_v_1.1, Na_v_1.2, Na_v_1.5, and Na_v_1.7 isoforms [91,92,93]. These dimers are linked with each other both directly and indirectly via specific proteins. The members of the dimers are not only physically attached, but the interaction results in coupled gating and function. First, co-expression of wild-type channels can help trafficking-deficient mutants to reach the plasma membrane. Second, it has been shown that some mutation can modulate the function of the non-mutated member in the dimer resulting in a dominant negative effect on the healthy channel [91,92]. This raises the possibility that if mutant non-cardiac VGSCs are present in the myocardium, they may negatively affect the function of normal Na_v_1.5 channels when they co-assemble. The notion of dimer channels is based on experimental data obtained in the expression system. It is essential to extend future studies to isolated cardiomyocytes in order to verify the existence of these phenomena in working cardiac cells.

### 3.3. Localization of Sodium Channels in Cardiac Myocytes and Potential Implications

Distribution of sodium channels shows a distinct pattern in the membrane of cardiac myocytes. Individual channels or doublets are arranged in clusters separated by channel-free membrane areas, and these clusters accumulate in the following membrane domains: intercalated disks, T-tubules, costameres, and caveola [55]. Cluster formation of sodium channels is not limited to cardiac myocytes; the phenomenon is reported in several other non-cardiac cell types as well [94,95,96]. It is very likely that channel clustering leads to functional consequences. Hichiri et al. demonstrated that sodium channel clustering facilitated the ephaptic conduction between neighboring cells altering the propagation of electric signals within the myocardium [97]. Bhargava et al. studied the localization of these clusters on the surface of rodent cardiomyocytes. They found that most patches contained 50 or less sodium channels, and channels accumulated on the crests between T-tubules [98]. The precise function of these clusters and the impact of clustering on late sodium current remains to be determined. The channel protein and current density is 3–6 times higher in intercalated disks than in the lateral membrane in rats. Interestingly, when the current is measured at the site of contact between two cells (in the intercalated disks between cell pairs), the current magnitude is larger than in disconnected state [99]. Based on the fine morphology of the perinexal membrane, Salvage et al. hypothesized that the interaction between α- and ß-subunits is different within the intercalated disks and the lateral membrane. They also proposed that the interaction between ß-subunits and cardiac or non-cardiac α-subunits are different. The ß-subunits belong to the cell-adhesion molecule family and serve as bridging units between the neighboring cells. They keep two sodium channels in opposing position within the intercalated disks increasing the probability for ephaptic conduction [100]. Nonetheless, potential interactions between the two pore-forming units have yet to be studied. Data regarding the distribution of cardiac and non-cardiac VGSC subtypes within the cardiac myocytes are somewhat conflicting. Lin et al. reported that non-cardiac VGSC was present in the lateral membrane but not in intercalated disk region in rats [99]. Verkerk et al. found the opposite situation in rabbits: TTX sensitive channels were localized primarily in intercalated disks in this species [101]. Recently, Struckman et al. reported that the neural sodium channel subtype Na_v_1.6 showed high accumulation in the T-tubule and was associated with Ryanodine receptors [102]. This observation was confirmed by Munger et al. in an independent study [103]. Ryanodine receptors are known to co-localize with L-type calcium channels (LTCC) and the Na^+^/Ca^2+^ exchanger (NCX) in cardiac cells [104]. This observation of Struckman et al. places cardinal players of the sodium and calcium homeostasis into the same micro-domain of the cardiomyocyte. Moreover, upregulation of late sodium current was shown in various heart diseases [15,25,26,27], and increased expression of non-cardiac sodium channels was reported in diseased myocardium [78]. The consequences of an altered proportion of cardiac and non-cardiac sodium channels on the sodium and calcium homeostasis of cardiac myocyte remains to be determined.

## 4. Role of Late Sodium Current in the Homeostasis of Cardiac Cell

The contribution of the late sodium current to the normal cardiac function is an important problem. Is this current a minor member of the set of cardiac ion currents, gaining importance only in pathologic conditions? The magnitude of late sodium current during the plateau is very small compared to the peak of the transient phase. Nonetheless, there are two facts to be considered in order to answer the question. First, following the upstroke of AP, major currents inactivate quickly; therefore, the plateau is maintained by a delicate balance of small currents. This increases the relative weight of the late sodium current. According to the evidence provided by Song et al., late sodium current is an important factor determining the length of the AP [105]. Second, the transient phase is short, undergoing full activation and inactivation within 2-5 ms. Therefore, the time sodium ions can pass through the membrane is limited. In contrast, the magnitude of late sodium current is less than 1% of the peak, but the time while it flows is much longer. In fact, late sodium current is ended only by the terminal repolarization of the AP. Consequently, despite the substantial magnitude differences between the peak and the late sodium current, the amount of the sodium entering into the cytoplasm is comparable [106,107].

### 4.1. Late Sodium Current and Sodium Homeostasis of Cardiac Cells

As discussed in the previous session, plateau sodium entry adds a substantial amount of sodium to the cytoplasm during electric systole. Increased late sodium current can elevate the cytosolic concentration substantially in diseased hearts. The cytosolic Na^+^ concentration changes dynamically with the heart function and is maintained by the balance of entry and extrusion [108,109,110]. The main route for sodium removal from the cytoplasm is the Na^+^/K^+^-ATPase (NKA) with stoichiometry of 3Na^+^/2K^+^ for the price of one ATP. The K_D_ for potassium and ATP are 80–150 µM and 2 mM, respectively; therefore, neither potassium nor ATP concentration are limiting factors for the pump due to their high concentrations [110]. In contrast, the K_D_ for Na^+^ falls close to the regular cytosolic sodium concentration with its value of 10–20 mM. Thus, NKA activity is highly sensitive to cytosolic sodium concentration. Hence, increasing cytosolic Na^+^ concentration facilitates NKA, thus catabolism. NKA is a major consumer of the ATP in cardiac cells, responsible for 9% of the total ATP utilization [111]. Considering that late sodium current upregulation occurs often in ischemic/hypoxic conditions, the facilitation of NKA might worsen the metabolic state of the cardiomyocytes, depleting the ATP content. Under these circumstances, NKA might fail to maintain the normal sodium concentration in the cytosol that leads to elevated sodium concentration in the cell [10,112].

### 4.2. Late Sodium Current and the Cardiac Calcium Homeostasis

The sodium and calcium homeostasis of cardiac cells is linked via NCX. Elevated cytosolic calcium concentration shifts the NCX equilibrium potential inhibiting reverse mode and facilitating forward mode. Thus, a fraction of the sodium is converted to calcium in the cytoplasm [6,111,113,114]. Ca^2+^ is the central regulator of cardiac cell function known to modulate metabolism, electric activity, contractility, development, and apoptosis [113,114,115,116,117,118,119]. Elevated cytosolic Ca^2+^ leads to calcium overload in the sarcoplasmic reticulum resulting in contractile dysfunction and arrhythmia. The direct link between altered late sodium current and calcium homeostasis has been demonstrated by several independent teams [49,52,120,121,122,123]. In a recent publication, Bengel et al. showed that Na_v_1.8 isoform might become a significant contributor to calcium homeostasis when late sodium current is upregulated [4,123]. This observation indicates the importance of non-cardiac sodium channels in the regulation of cardiac function. The significance of the cytosolic sodium concentration in modulating the cardiac cell calcium homeostasis is discussed in a paper by DW Hilgeman [124]. According to the analysis of the author, when free sodium concentration increases from 5 mM to 12 mM in the cytoplasm, the diastolic calcium concentration grows from 20 nM to 300 nM. Considering the steep dependence of contractility on the cytoplasmic calcium concentration (Hill slope is as high as 5 in cardiac myocytes), a few mM changes in sodium concentration must have a substantial impact on the myocardial pump function.

The calcium ion is the central regulator of metabolic enzymes in various cell types including cardiac myocytes. Many mitochondrial enzymes are stimulated by the oscillating calcium concentration during the systole. The altered calcium homeostasis may result in retuning of the complex metabolic machinery of the cardiomyocytes referred as metabolic remodeling [125].

## 5. Electrophysiology of Late Sodium Current

VGSCs play a key role in the activation of cardiac myocytes and the electric signal propagation within the myocardium. The positive feedback coupling between the membrane potential and sodium channels initiates the transition from closed to open state of the channels within a few milliseconds, followed by rapid inactivation. Hence, 3–5 milliseconds after the upstroke of the AP, 99% of sodium channels are in an inactivated, non-conductive state. This narrow spike of the current is termed the transient phase. Nevertheless, a small fraction of channels fail to inactivate, maintaining a small sustained current during the AP plateau referred to as late sodium current. The particular details of how this small fraction of channels avoids inactivation are not understood, and several mechanisms are proposed in the literature.

### 5.1. The Window Mechanism

Window theory is usually the first mechanism authors mention to explain the origin of late sodium current in most publication discussing the topic [15,23,24,126]. The window phenomenon is not specific to VGSCs, as it is well known in calcium and potassium channel electrophysiology as well [126,127]. Channels may recover from inactivation then reopen at this voltage range repetitively. The resultant oscillation between conducting and non-conducting states can maintain a steady-state current as long as the membrane potential is held within the window range. This flip-flopping may explain how the relatively fast activation and inactivation kinetics of VGSCs can maintain a persistent current present during the whole AP plateau. Despite its plausibility, the contribution of the window mechanism to late sodium current might be limited by two factors. First, the maximum availability of the channels at the crossing point is less than 5% [87,128,129,130]. Second, the low availability is further reduced by the substantial voltage difference between the AP plateau and the window range. The center of the window range falls to around −60 mV at cardiac isoform (Na_v_1.5), while the AP plateau is found well above zero mV in most mammalian AP. This difference markedly reduces the probability for opening in most species [128,131]. Experimental data presented by Beyder et al. indicate that mechanical stress shifts the window toward negative voltages causing further reduction in channel availability [132]. Nevertheless, pathologic conditions may modify the configuration of the window to directions that favor the facilitation of late sodium current. Increased cytosolic calcium concentration [133,134], mutations [133,135,136,137,138], or the presence of non-cardiac isoform [4,81,139,140] might shift either the steady-state inactivation or activation curve altering the position or the size of the window, hence increasing its contribution to late sodium current.

### 5.2. Sodium Channel Gating Modes

Cardiac AP starts with a rapid depolarization, initiating a sequence of conformational changes in VGSCs. Sodium channels open and inactivate within two-four milliseconds, then they may produce up to 10 rapid reopening cycles before finally getting absorbed in the inactivated state [139,140,141]. If the flickering of the channel is terminated within 40 milliseconds, the mode of activity is termed “transient mode.” Transient channel activity is the mayor form of channel activity during the sodium current peak. It results in a quick rise of the ensemble current followed by a rapid decline in the whole cell. The transient mode contributes with less than 1% to the total current 20 milliseconds after the peak. A small fraction of channels fail to inactivate and maintain sustained activity while the membrane is depolarized. The activity pattern of these non-inactivating channels could not be distinguished from the rest of the channels during the transient phase. Their identification is based on the fact that their activity is maintained over 20 milliseconds. Two activity modes were observed in this phase termed as “burst mode” and “late scattered mode.” Channels in burst mode display long-lasting (100–300 ms), rapid flickering between conducting and non-conducting states terminated by inactivation. These non-inactivating bursts have already been reported from both skeletal and cardiac muscle and were referred to as “cloudburst” currents [142,143,144]. The burst activity is a rare event, observed in less than 0.1% of channels, but certain chemicals are reported to increase its occurrence [145]. The contribution of burst mode to the peak current is negligible, but as transient mode activity declines, it can grow as high as 50% of the total current. Burst mode activity then diminishes, and 200–300 ms later it is replaced by “late scattering mode.” This third mode of channel activity is similar to the transient activity, but non-conducting states are longer between two brief openings, and terminal inactivation occurs after 500–1000 milliseconds.

The three different activity modes follow each other in a timed sequence during the AP. The first five milliseconds following the upstroke is dominated by the transient mode, and the relative contribution of the other two operation mode is negligible. This is followed by an intermediate phase (5–40 ms) when all three modes are active with a rapidly declining weight of the transient mode. There is no clear margin line between the transient and late phase of sodium current, but most researcher puts the boundary between 5–20 milliseconds following the AP upstroke. The contribution of burst mode and late scattering mode is similar in this phase, then the monotonic reduction of burst mode leaves the late scattering mode the only type of channel activity after 100–150 milliseconds. Changes in the relative contribution of the different activity modes to the total sodium current are implicated in cardiac dysrhythmias, and the pharmacological modulation of these operation modes is hoped to exert beneficial effects in various heart diseases [12,13,59,78,87,128,146,147,148,149,150,151,152].

### 5.3. Non-Equilibrium Gating

This interesting mechanism was proposed by Clancy et al. in 2003 [44]. The basic concept is based on the assumption that the transition rate between various states is modulated by the voltage trajectory previously experienced by the channel. According to the proposed mechanism, the channel’s recovery from inactivation is facilitated by repolarizing ramps. This condition is present during the plateau of cardiac AP but not in traditional voltage clamp experiments where rectangular voltage commands are used. The non-equilibrium gating hypothesis was confirmed by several observations. In their article Clancy et al. published the hypothesis and provided convincing experimental and in silico data to support their theory. First and foremost, using an expression system, they demonstrated a hump on the sodium current during hyperpolarizing ramp. The hump occurred more than 100 milliseconds after the transient phase, and the peak was seen at −20 mV, which is outside of the window range. Additional supporting experimental evidence was provided by Magyar et al. who demonstrated that the open probability of sodium channels is higher during ramp command than at constant voltages, and the duration of the sodium current is influenced by the parameter of the ramp [153]. Furthermore, Horvath et al. showed that the late sodium current magnitude is greater during the AP plateau of that seen during square pulse commands [1]. It is interesting to note that late sodium current displayed a hump on their records between 0 and −20 mV, like that reported by Clancy et al. [44]. Recently, two interesting observations were published by An Tuan Ton et al. supporting the non-equilibrium gating mechanism [54]. First, they confirmed the facilitating effect of the downward ramp on late sodium current in primary human ventricular myocytes. Second, the peak of the hump recorded fell between 0 and −20 mV much like those seen by Clancy et al. and Horvath et al.

These observations indicate that non-equilibrium gating is an existing and chief factor in determining the magnitude and the profile of late sodium current. This mechanism does not preclude the contribution of other mechanisms like window theory or different gating modes to late sodium current. These mechanisms might coexist and shape late sodium current in synergism during the AP. Nevertheless, we can assume that different gating modes probably have different drug sensitivities or affinities [52,154,155] and understanding these molecular events can help to develop new antiarrhythmic strategies.

### 5.4. Resurgent Current

**Figure 1:**
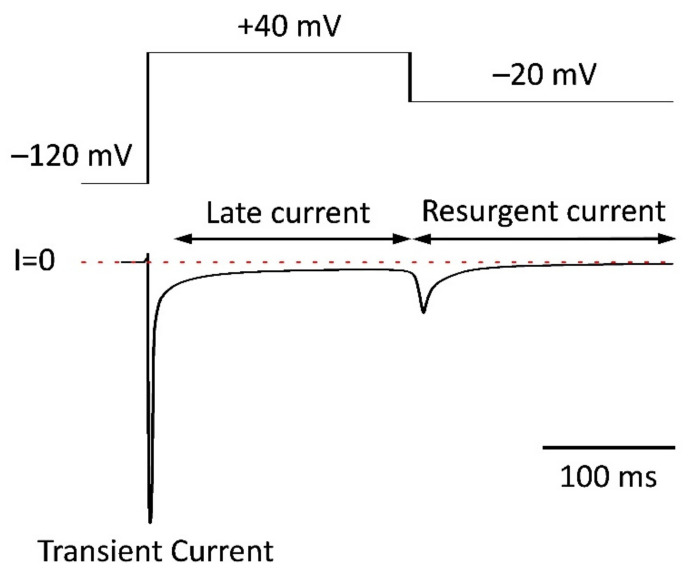
Schematic representation of the transient, late, and resurgent phases of sodium current during a rectangular command pulse. The transient phase is activated by brief depolarization, reaches the peak within a few milliseconds, and decays rapidly. The late phase follows the transient phase and terminated by the repolarizing step. The repolarization step activates the resurgent current.

The resurgent current results from a specific way process of channel reopening reported first from cerebellar Purkinje neurons [156]. The proposed mechanism involves an ultra-fast open-channel block mediated by the C-terminus of ß4 subunit during the depolarization and followed by a repolarization induced rapid recovery from inactivation. This recovery gives rise to a tail current during the repolarization step (Figure 1), before the channel is absorbed in the regular inactivated state, resulting via occlusion of the pore by the linking region between DIII and DIV [90,154]. According to the suggested mechanism, the C-terminus of the ß4 subunit and the inactivation particle of the channel competes for the binding site in the pore, and repolarization favors the dissociation of the ß4 subunit reopening the pore. The separation of the resurgent current from late sodium current is easy when rectangular voltage command is used because the late sodium current is seen during the depolarization, while the resurgent current starts with the repolarizing step. However, the two activity modes may coincide during ramps or AP plateau. The presence of resurgent current was demonstrated in Na_v_1.4, Na_v_1.5, Na_v_1.6, and Na_v_1.7 based sodium channel expression models and neurons, but not in cardiac myocytes [157]. Since mutations of neuronal sodium channels are known to facilitate resurgent current and expression of neuronal VGSCs is demonstrated in cardiac myocytes, the resurgent current may potentially contribute to late sodium current and arrhythmogenesis. Various toxins are reported to facilitate resurgent currents, including ß-pompilidotoxin and ß-scorpion toxin [155,158], raising the possibility that pathologic regulation or pharmacological modulation of VGSCs may increase the contribution of resurgent current to cardiac late sodium current. The contribution of resurgent current to cardiac late sodium current has been left essentially unexplored. Further work is needed to determine whether this mechanism has any relevance in cardiac pathophysiology or not. It is an important question as to whether the mediation of ultra-fast inactivation is exclusive to ß4 subunits or if other subtypes can promote it too. In their review article, DeMarco & Clancy propose that non-equilibrium gating and resurgent current share the same mechanism [159]. Nonetheless, the non-equilibrium hypothesis has been derived from experimental data obtained in an expression model that expressed no ß4 subunit. There is a substantial amount of research work to be done on this area.

## 6. The Modulation of Late Sodium Current

Adapting the activity of cardiac myocardium to changing conditions requires precise control of ion channels on a moment-to-moment basis. VGSC, due to its pivotal role in excitability, is a target of various signaling pathways in the cardiac myocytes. These modulatory pathways exert their effect simultaneously and in a synergistic manner.

### 6.1. The Calcium—Calmodulin—Calmodulin Kinase Axis

The calcium—calmodulin (CaM)—calmodulin-dependent kinase (CaMK) axis is probably the most studied part of the VGSC regulation. These elements are shown to regulate VGSC individually and cooperatively [115,160]. Despite the increasing body of experimental data on the subject, we are only beginning to understand the complex regulation exerted by Ca^2+^-CaM-CaMK system on late sodium current. Although there are substantial differences in the fine details among various experimental models, the activation of the whole Ca^2+^-CaM-CaMK pathway seems to facilitate late sodium current in most species [115,161].

#### 6.1.1. Direct Regulatory Effect of Ca^2+^ on VGSC

The calcium ion was demonstrated to bind to a dedicated motif termed “EF hands” located close to the C-terminus and modulate channel function in VGSCs [134]. The “EF hands” motif is consistently present in hundreds of proteins regulated directly by Ca^2+^. According to our current understanding, this structure binds Ca^2+^ with high selectivity [162]. Therefore, the presence of this motif within the channel protein strongly supports the hypothesis that Ca^2+^ exerts a direct control on VGSCs. Wingo et al. provided numerous experimental data supporting this hypothesis [134]. First, the binding of Ca^2+^ to the “EF hands” has been demonstrated with NMR spectroscopy. Second, the steady state inactivation curve of the VGSCs is shifted toward positive voltages in high cytosolic Ca^2+^ concentration even in the presence of the CaM inhibitor peptide. Third, mutations within the “EF hands” region were shown to prevent both Ca^2+^ binding and Ca^2+^ induced shift of the SSI curve. These experimental observations corroborate the direct controlling theory of Ca^2+^. Nonetheless, several teams challenged this theory questioning the efficacy of CaM inhibiting peptide used by Wingo et al. Opponents of the direct Ca^2+^ control theory hold that CaM is essential to mediate the effect of Ca^2+^ on VGSCs [163,164,165]. The apparent conflict between the two theories could be resolved by the model presented by Shah et al., which attempts to combine the two concepts [133]. According to the proposed hypothesis, the inactivation is modulated by the interaction of two motifs within the channel, namely the “EF hands” and IQ. During diastole, when cytosolic Ca^2+^ concentration is low, CaM binds to the IQ motif preventing the interaction with “EF hands.” When cytosolic Ca^2+^ concentration is elevated, CaM binds Ca^2+^, reducing its affinity to the IQ motif. Then the Ca/CaM complex dissociates from the IQ segment unmasking the binding site and allowing the interaction with the “EF hands.” According to the hypothesis, the interaction between the two segments increases the calcium affinity of the “EF hands.” In this manner, the regulatory role of Ca^2+^ and CaM are combined. Later, Biswas et al., using truncated mutants, reported that the high Ca^2+^ could exert its regulatory effect in sodium channels that lack the IQ motif [166].

#### 6.1.2. Calmodulin

CaM mediates calcium effects to target peptides in various cell types including cardiac myocytes [164,165]. CaM is a small protein containing 148 amino acids, with two globular ends (the N- and C-lobes) connected by a flexible linker region. Both N- and C-lobes contains two “EF hands” motifs allowing CaM to bind as many as four Ca^2+^. CaM forms a bridge between the IQ motif and the DIII-IV linker region of the VGSC [167]. This flexible linker region is known to function as the inactivation gate of the sodium channel [168], and CaM is known to modulate channel gating [167,168,169]. At low cytosolic calcium concentrations, N- and C-lobes lack calcium (apo-CaM) and the C-lobe masks the IQ motif of the channel preventing interaction with the DIII-IV linker region [134,170,171]. At high calcium levels, the calcium-CaM complex is formed (holo-CaM), and the C-lobe dissociates from the IQ motif due to its reducing affinity [133]. Furthermore, the apo-holo transition induces profound conformational change in the CaM, altering its orientation as well. The holo-CaM binds to the IQ motif through the N-lobe allowing C-lobe to interact with the DIII-IV linker region resulting in a shift in SSI curve [167]. CaM is also shown to bind to the N-terminal domain of sodium channels. Wang et al. reported the presence of a CaM binding domain on the N-terminus of VGSCs, similar to that known from voltage gated calcium channels [172]. According to their observations, deleting the amino acids 80–105 abolished the interaction between the N-terminus of the sodium channel and CaM in the expression model. The affinity was reduced by certain mutations in the N-terminus as well. Based on their data, Wang et al. raised the possibility that the interaction between CaM and the N-terminus may control the dimerization of sodium channels. Additionally, CaM is known to activate CaMK that serves as a sovereign signaling pathway modulating VGSC function [173].

### 6.2. Protein Kinases

There are numerous phosphorylation sites on the α-subunits of the VGSCs. The individual residues are targeted by various kinases, and phosphorylation is known to modulate diverse channel functions [169,170,171,174,175].

#### 6.2.1. Calmodulin Kinase

CaMK is a serine/threonine kinase that plays a pivotal role in the regulation of numerous cell functions in various cell types including cardiac myocytes. Up until now, more than 15 members of the CaMK family have been identified with numerous splice variants. Cardiac cells express two isoforms of the type II CaMK named δ_B_ and δ_C_, referred to often as nuclear and cytoplasmic isoforms. Sodium channels are phosphorylated by CaMKIIδ_C_ [113,114,115]. Experimental data show that CaMKIIδ_C_ targets specific serine (571, 483, 484, 516) and threonine (594) residues leading to changes in gating kinetics and current magnitude [169,176,177]. While considerable species differences are reported on channel gating, it seems that the overall effect of CaMKIIδ_C_ is increasing the magnitude of late sodium current in every model. Consistently, the inhibition of the enzyme leads to the reduction of late sodium current. The overexpression of the enzyme was reported to cause a negative shift of the SSI curve in rabbit hearts [178]. Similar observations were made in the expression system by Ashpole et al. and Koval et al. [179,180]. In contrast, a positive shift was reported by Aiba et al., who used freshly isolated guinea pig ventricular cells, and CaMKIIδ_C_ was added to the pipette solution [181]. According to numerous reports, the facilitation of CaMKIIδ_C_ consistently induced no change in the voltage dependence of activation [169,176,177,182]. Increased peak amplitude for the transient phase was reported by Aiba et al., in the presence of high CaMKIIδ_C_ concentration [181], others observed no change in this parameter [178,179,180]. Available data are limited regarding the modulation of the inactivation dynamics of the sodium current. Wagner et al. reported significant deceleration of the decay phase [178], but Aiba et al. detected no change [181]. In contrast to the diverse data regarding the transient phase of sodium current, it is widely accepted that CaMKIIδ_C_ facilitates late sodium current via enhancing the fraction of channels undergoing intermediate or slow inactivation. In line with this, CaMKII inhibitors are shown to reduce late sodium current [1]. This experimental observation was confirmed by others in both healthy and diseased models [165,183,184]. Hegyi et al. employed an action potential clamp technique to study the role of CaMK on late sodium current in freshly isolated rabbit ventricular myocytes. According to their observations, late sodium current had a basal level partially dependent on CaMK. CaMKII activation facilitated late sodium current during the late phase of the plateau [2]. To the best of our knowledge, this is the first publication reporting different CaMK sensitivity in different parts of the late sodium current during the action potential.

#### 6.2.2. Protein Kinase A (PKA)

This enzyme is a downstream effector of the ß-adrenergic receptor—G protein—cAMP pathway regulating multiple functions in cardiac cells. Due to the broad scale of its intracellular targets, the overall effect on late sodium current is debated [181,185]. Further complication arises from the fact that ß-adrenergic receptor activation ignites other signaling pathways in the cell, including CaMK, NOS, and ROS [186]. PKA is shown to facilitate sodium channel trafficking, hence increasing the channel density in the sarcolemma, hence, the peak current [176,177]. According to Tateyama et al., late sodium current is insensitive to PKA when measured in wild-type sodium channels expressing HEK cells. On the contrary, PKA increased the sustained component of sodium current in D1790G mutant channels [187]. Hegyi et al. studied the regulatory effect of PKA on late sodium current during AP in isolated rabbit ventricular cells. They showed that PKA had no contribution to the basal level of late sodium current. However, when PKA was stimulated in these freshly isolated cells, the current increased significantly during the early plateau [2]. Recently Fouda & Ruben addressed the problem and demonstrated that PKA activator CPT-cAMP exerted a strong stimulatory effect on late sodium current, increasing the amplitude to more than twice the control value in cultured cells. The specific stimulatory effect was verified by using PKA inhibitor H-89 [182].

#### 6.2.3. Protein Kinase C (PKC)

At least seven PKC family members are identified in mammalian hearts, but the expression level of the various isoforms show big interspecies differences [183]. Most isoforms are activated by Ca^2+^, making PCK a parallel signaling pathway for CaM and CaMK [184]. The serine residue phosphorylated by the enzyme is found in positions 1503 and 1505 in human and rodent VGSCs, respectively. The phosphorylation of the channel protein at this position shifts the SSI curve and alters the inactivation [171,185]. The direction and magnitude of the SSI shift is different in various models. Ma et al. reported the facilitation of late sodium current following PKC activation in rabbit hearts [188]. Contrarily, Qu et al. observed voltage dependent reduction of the peak amplitude with negative SSI shifts in the Xenopus oocyte expression model [189]. Single channel data showed reduced early (t < 5 ms) and late (t > 10 ms) opening probabilities following PKC activation. The conflicting data could be explained by the different experimental model. In the Xenopus oocytes used by Qu et al., ß-subunits were not expressed. Nonetheless, Ma et al. used freshly isolated cardiomyocytes with intact channels. Based on these conflicting observations and their experimental data, Ashpole et al. proposed an interesting working model. According to their hypothesis, the regulatory effects of sodium channel phosphorylation cannot manifest without the presence of regulatory proteins, like ß-subunit [179]. Recently, Fouda & Ruben reported that PKC activation doubled late sodium current magnitude in expression system [182].

#### 6.2.4. Serum—And Glucocorticoid-Inducible Kinases (SGKs)

Three isoforms (SGK1, SGK2, and SGK3) of the enzyme were identified from various tissues including heart and tumor cells [190,191,192]. The dominant isoforms in heart are SGK1 and SGK3. SGK2 is expressed in a lower amount [193]. The enzyme is activated by circulating hormones (insulin, insulin-like growth factor, and glucocorticoids) and oxidative or metabolic stress [190,193]. Increased SGK activity was reported from various diseases, including heart failure, cardiac fibrosis, liver cirrhosis, or nephropathy [194,195,196]. Upregulation of SGKs was shown to facilitate hypertrophic response and suppress apoptosis in cultured cells [190]. SGKs were shown to stimulate sodium current in various ways [197,198]. Increased window current caused by leftward shift of activation and rightward shift of inactivation curves was observed in the Xenopus expression model [198]. These results were partly confirmed in mice, where both inactivation and activation curves (and the crossing point) were shifted toward negative voltages following SGK stimulation [197]. SGK was found to increase channel availability and current density in these reports [197,198]. The postulated mechanism involves reduced binding of ubiquitin ligase Nedd4-2 to PY motif of VGSC. These observations point to the direction that SGKs could play a central role in the modulation of sodium channels in the heart. Further experimental data seem to corroborate this hypothesis. First, cortisol was demonstrated to modulate VGSC expression in fetal sheep myocardium [199]. Second, according to the observations of Das et al., late sodium current was increased markedly in transgenic mice with constitutively active SGK1. This increased late sodium current coincided with prolonged AP and increased propensity to ventricular arrhythmias. Importantly, ranolazine, the selective late sodium current inhibitor, was demonstrated to alleviate both AP lengthening and pro-arrhythmic effects of increased SGK1 activity [197].

### 6.3. Metabolic Control

Cardiac force generation and metabolic activity adapts on a moment-to-moment basis according to the fluctuating demand on the heart caused by changing peripheral resistance, physical activity, and emotional state. The metabolic state of the cardiac myocyte is known to modulate the electric activity of the sarcolemma via numerous feedback mechanisms altering ion channel and pump functions. VGSCs are shown to be sensitive to cytosolic pH and metabolites. The pH of the cytosol in myocardial cells may decrease as low as 6.0 during hypoxia [200], which is known to have robust impact on the channel function [194,195,196,201,202]. Acidosis was reported to shift the voltage dependency of both inactivation and activation curves in a positive direction and slow down the inactivation of the transient phase [196,201,202]. Jones et al. reported increased window current in the Xenopus model and predicted a lengthening of AP using computer simulation [201]. These observations were partly confirmed in freshly isolated canine cells by Murphy et al. [194,195]. They found a depolarizing shift in the voltage dependency of activation but not in the steady state inactivation curve. They successfully demonstrated the AP lengthening predicted by Jones et al., but the late sodium current was found to be reduced in both endocardial and epicardial myocytes [194].

Cardiac myocardium is very sensitive to reduced oxygen supply, and hypoxia has been linked to electric disturbances and arrhythmia in the heart. There is a consensus on the notion that hypoxia facilitates late sodium current contributing to arrhythmogenesis [45,203,204,205,206,207]. Wang et al. studied the mechanism of hypoxia-induced late sodium current facilitation and observed increased burst activity in a single channel model after 15 min in a hypoxic milieu [207]. This increased burst activity may explain the increased late sodium current. Interestingly, they found a reduced peak and leftward shift in the voltage dependence of the SSI curve. According to our current understanding, the leftward shift of the SSI curve should have attenuated the facilitation of late sodium current due to the reduced window current. These data may raise the possibility that the contribution of window current to late sodium current is limited. Furthermore, Wang et al. reported a shortening of cardiac AP under hypoxic conditions, which may indicate that other hypoxia sensitive ion channels are present in the myocardium. Recently, Plant et al. demonstrated that hypoxia increases the SUMOylation of the sodium channels in human pluripotent cells. This SUMOylation is necessary and sufficient for hypoxic facilitation of late sodium current [208].

Hydrogen peroxide and free radicals are demonstrated to facilitate late sodium current [10,209,210,211]. In accordance with this, TTX or specific late sodium current inhibitor ranolazine is shown to attenuate the AP lengthening effect of H_2_O_2_ [211]. Since CaMK is known to be directly activated by free radicals [196], to understand the fine details of the complex redox control of late sodium current, further exploration is needed.

A broad scale of second messengers and metabolites are known to modulate late sodium current. Docosahexaenoic, eicosapentaenoic, and other poly-unsaturated fatty acids were shown to reduce both peak and late sodium current due to leftward shift of SSI and activation curve reducing window current [212]. Lysophosphatidylcholine is an ischemic metabolite was also found to reduce the magnitude of the transient phase [46,213]. Regrettably, late sodium current was not studied in these studies. Nitric oxide was found to facilitate late sodium current by Ahem et al. They proposed that nitrosylation can modify the gating of sodium channels [214]. Later, this hypothesis was confirmed by Cheng et al., who also demonstrated that caveolin-3 mediated the channel nitrosylation [215]. Because cardiac CaMK, the pivotal regulator of sodium channels, is also known to be modulated by nitrosylation, these interactions could be complicated with several parallel mechanisms [216].

Recently, Matasic et al. reported that NAD^+^ precursor, nicotinamide riboside, increases the peak but reduces late sodium current [217]. According to their report, nicotinamide riboside exerts its different effects via multiple mechanisms including inhibition of deacetylation of Na_v_1.5 and PKC activation.

### 6.4. Mechanical Stress

Myocardial wall tension changes on a moment-to-moment basis during the cardiac cycle and all proteins in the sarcolemma, including ion channels, are subjected to varying mechanical stress. There is a consensus that VGSCs, like other ion channels, respond to mechanical stress with altered kinetics [132,218]. Beyder et al. reported a negative shift of both SSI and activation curves and slower inactivation of the sodium current during mechanical stress in cultured cells [132]. At the same time, the availability of the channel was increased, resulting in increased current magnitude. Later these findings were confirmed in freshly isolated mouse ventricular cells [219]. The authors also reported that ranolazine reduced the mechanosensitivity of cardiac sodium channels (Na_v_1.5) in a dose dependent manner. This observation was confirmed by the same team in cultured cells [220]. Ranolazine is an antiarrhythmic drug inhibiting late sodium current with high selectivity over the transient phase [20,51,52,154,221]. Considering that myocardial wall stretch has been linked to clinical arrhythmias for a long time [222,223,224], these data may help to develop new therapeutic strategies in the antiarrhythmic pharmacology. Currently, myocardial wall stress is reduced in clinical practice by reducing central venous pressure with diuretics. Reducing the mechanical sensitivity of the electric machinery of the sarcolemma could prove to be useful as supporting therapy in certain types of arrhythmias.

### 6.5. Accessory Proteins

Several proteins have been shown to interact with the pore-forming subunit of VGSC, including the ß-subunits [225,226,227], small G-proteins [228], Ankyrin G [221], SAP97 [229], and ubiquitin [230]. Accessory proteins fulfill various functions during the life cycle of sodium channels being partners in posttranslational trafficking, supporting the anchoring at the target position, modulating gating properties, and controlling channel internalization/degradation. Some of these proteins, such as ß-subunits or ubiquitin, have been the subjects of intensive research for a long time, but others, like small G-proteins and Ankyrin G, have only recently attracted attention. Mutations in some of these accessory proteins have already been linked to various cardiac diseases [85], and we can assume that further harmful mutations are to be uncovered. Recently, there was a boom in this field, resulting in numerous publications. Due to volume limit, we refer to excellent reviews published recently [221,231].

## 7. Pathologic Aspects of Late Sodium Current Function

Upregulation of late sodium current has been linked to pathologic cardiac function including arrhythmia, contractile dysfunction, and structural heart diseases for a long time [12,16,62,232,233,234]. Numerous pathologic conditions such as mutations, hypoxia, toxins, and the upregulation of CaMK are known to facilitate late sodium current leading to cardiac dysfunction [16]. There are two possible ways to upregulate late sodium current in the heart. First, increasing the expression of cardiac and/or non-cardiac isoforms, and second, altering channel gating. The increased expression of cardiac and non-cardiac isoforms were reported from remodeled myocardium. Remodeling can be induced by structural heart disease, pressure/volume overload, or myocardial infarction [78,113,235,236,237]. Altered channel gating was identified in various clinical conditions and disease models [45,148,188,205,207,238]. There are cases when late sodium current facilitation results from the combination of multiple pathologic factors. Late sodium current upregulation caused by remodeling induced increased expression and hypoxia-induced altered gating often combines in various heart diseases [113,114]. Oddly, increased late sodium current magnitude coinciding with reduced expression of Na_v_1.5 is also reported in the literature [14].

Acquired or inherited increase of late sodium current is associated with an enhanced risk for cardiac arrhythmia, and inhibition of late sodium current was demonstrated to exert beneficial effects [15,16,20,23,49,62,123,153,239].

### 7.1. Arrhythmias

Arrhythmic activity may develop via multiple mechanism when late sodium current is upregulated.

First, upregulation of late sodium current was shown to lengthen AP, increasing the risk for early afterdepolarization (EAD). EADs are slow membrane potential oscillations caused by the reactivation of depolarizing currents during phase two or three of the AP. They are implicated in triggered arrhythmias [239,240]. The possible candidates for reactivating currents are late sodium current, I_Ca,L_ and I_NCX_. Calcium overload was documented to promote EADs, but the mechanism is not completely understood [241,242]. It has been proposed that spontaneous calcium release from the sarcoplasmic reticulum may contribute to EADs via facilitation of NCX [243,244,245]. Horvath et al. addressed the contribution of late sodium current to EADs in an interesting study [1]. They showed that facilitation of late sodium current with ATX II prolonged AP duration and induced EADs coinciding with cytosolic Ca^2+^ oscillations. When Ca^2+^ oscillations were suspended with BAPTA, the AP remained long but EADs were terminated. Based on these observations the authors proposed that increased late sodium current may contribute to the Ca^2+^ overload of the sarcoplasmic reticulum, but EADs arise from spontaneous calcium release from intracellular stores.

Second, increased late sodium current was associated with Delayed Afterdepolarizations (DADs) too. DADs arise from resting membrane potential during electric diastole and are explained by facilitation of NCX by spontaneous calcium release from the sarcoplasmic reticulum [149,246]. The mechanism is similar to that of EADs; late sodium current provides no depolarizing power, and its role is limited to induce calcium overload [16,247,248,249,250].

Third, upregulation of late sodium current is associated with increased beat-to-beat variability and regional inhomogeneity of AP duration [41,217,251,252,253]. Increased beat-to-beat variability results from reduced repolarization reserve. It lengthens AP duration increasing the risks for EADs and triggered activity [254]. Regional and transmural differences in AP duration are generally attributed to an asymmetrical distribution of various ionic channels [243,244,245,251,255,256,257]. Increase in both beat-to-beat variability and transmural heterogeneity may serve as a substrate for arrhythmic activity due to the increased AP dispersion under pathologic conditions [258,259].

Fourth, increased late sodium current but not peak amplitude is reported from atrial fibrillation (AF) in humans [14]. AF is the most prevalent form of cardiac arrhythmias [260,261], and is known to cause electric remodeling of the myocardium that leads to downregulation of calcium and potassium currents and shortening of AP duration [252,253].

Fifth, various mutations of VGSCs are associated with different forms of Brugada syndrome and Long QT syndrome [138,262,263,264,265,266]. These pathologic states are characterized by altered heart rhythm, impulse conduction, and repolarization. The clinical diagnosis is based on ECG findings. Both disorders are subject of intensive research; the popularity arose in part from the fact that the multilevel connection between the genetic defect and the clinical symptoms is exceptionally well established in these two cardiac diseases.

### 7.2. Late sodium Current and Dilated Cardiomyopathy

Dilated cardiomyopathy (DCM) is a progressive structural heart disease characterized by dilated chambers and reduced myocardial force generation. The first observations that linked *SCN5A* mutation to DCM were published in 2004 and 2005 by two independent research team [267,268]. The proposed hypothesis that the mutation of a channel gene and the resultant channel dysfunction may induce structural heart disease raised doubts in the scientific community. The hypothesis was challenged by Groenewegen & Wilde who suggested another gene responsible for DCM phenotype [269]. Continued research identified new, previously unknown mutations in the *SCN5A* gene of DCM patients providing further evidence for the idea that morphological heart disease may develop on the base of sodium channelopathy [270,271]. Despite the growing body of supporting evidence, the mechanism of how a mutant channel can cause structural heart disease was not clarified. The explanation came from Gosselin-Badaroudine and his co-workers in 2012. They have shown that the mutation made the sodium channel permeable for protons through an alternative pore not identical to the sodium path [272]. According to their proposed model, the persistent inflow of protons resulted in the acidification of the cardiomyocyte’s cytoplasm, leading to the DCM phenotype of these patients. The association between the SCN5A mutation and DCM has been confirmed by numerous publications, subsequently [273,274,275,276,277].

## 8. Pharmacology of Late Sodium Current

This topic has been addressed by several articles lately, and the list of drugs is available in a number of reviews [25,26,27]. Thus, we limited ourselves to a review of some of the most recent developments.

### 8.1. Eleutheroside B, a New Late Sodium Current Inhibitor

This drug is the main constituent of the Chinese herb, Acanthopanax senticosus, known as Siberian ginseng. The drug has been used in traditional Chinese medicine to treat various diseases including inflammation, tumors, and diabetes. Clinical observations reporting potential cardioprotective effects motivated researchers to test the drug on rabbit atrial myocytes [278]. The authors reported dose-dependent inhibition of late sodium current following the application of the drug in both control and ATX II stimulated cells with IC_50_ = 167 µM. The drug reduced peak current at higher concentrations as well. Calcium and potassium currents were found insensitive to the drug. The drug prevented the ATX II induced elevation of both systolic and diastolic calcium concentration in the cytoplasm. The authors presented further supporting data obtained in Langendorff heart.

### 8.2. Old Drugs with New Therapeutic Effects

Empagliflozin, dapagliflozin, and canagliflozin [122] are type two sodium/glucose cotransporter inhibitors used in the therapy of diabetes are known to exert significant cardioprotective effects. Philippaert et al. hypothesized that the cardioprotective effect might be in part caused by a direct cardiac effect. The drugs tested exerted a robust inhibitory effect on late sodium current without altering the peak in various experimental conditions, including pressure overloaded cardiomyopathy, LQT3 mutant, and the following H_2_O_2_ stimulation. Empagliflozin was found to improve the calcium handling of isolated cardiomyocytes in veratridine induced arrhythmia. This interesting study may open a new direction to identify new late sodium current inhibitors.

Riluzole [103,279], a neural sodium channel blocker has effectively been used to manage amyotrophic lateral sclerosis in clinics. Munger et al. postulated that inhibition of the neuronal sodium channels may prevent atrial fibrillation. They addressed the problem in both animal experiments and post hoc clinical data analysis. Using pathologic mouse and canine models they demonstrated that riluzole effectively inhibits late sodium current, suppresses aberrant calcium oscillations, and exerts beneficial effects in arrhythmia. Clinical data revealed that the arrhythmia risk of riluzole-treated patients were lower than that of the control group. They concluded that riluzole has the potential to be repurposed as a therapeutic tool for preventing atrial fibrillation.

### 8.3. ATX-II. New Observations with an Old Tool: Friend or Foe?

ATX-II is a popular pharmacologic tool to amplify late sodium current in both cardiac and non-cardiac cells [10,280,281,282]. The magnitude of this tiny current barely exceeds the peak-to-peak value of noise in most cell types including, neurons or isolated cardiac myocytes. Researchers often employ ATX-II to amplify the current. The toxin augments late sodium current significantly, thus improving the signal-to-noise ratio and increasing the reliability of measurements substantially. Potential late sodium current inhibitors or other drugs are often tested against this toxin [8,10,152]. A comprehensive study on the mechanism of electrophysiological effects of ATX-II on sodium channels has never been published, but sparse data are available. ATX-II was shown to shift the voltage dependence of the steady-state inactivation and activation curves [281,283] and alter recovery from inactivation [8]. Recently Ton et al. reported interesting observations on the ATX-II effects on sodium current in the human heart [54]. According to their data, ATX-II resulted in a significant negative shift in the voltage-current relationship. Similar observations reporting moderate shifts were published earlier in neurons [284]. These reports indicate that ATX-II not only boosts the current magnitude but alters the voltage dependency of several parameters. Consequently, caution must be exercised when experimental data obtained in ATX-II treated cells are interpreted.

## 9. Summary and Perspectives

Late sodium current has been the focus of research for a long time. Since the first reports on the persistent sodium current, significant progress has been achieved, especially in the last two decades. The knowledge we have accumulated has helped us to understand various aspects of the physiologic and pathologic role of late sodium current better, and new therapeutic paradigms were established resulting in new antiarrhythmic drugs. Still, our understanding is far from complete. There is a consensus that increased late sodium current deteriorates the normal electric activity and sodium/calcium homeostasis of the heart. The scientific society is in agreement that reducing the pathologically enlarged late sodium current is beneficial for the diseased heart. However, the particular details of the contribution of late sodium current to cardiac AP and sodium homeostasis have not been explored.

One of the greatest limiting factors in late sodium current research currently is the lack of specific inhibitors for the different isoforms and gating modes. The absence of these specific pharmacologic tools hampers the unfolding of the complex interplay among the different types of sodium channels. Another limiting factor is the enormous complexity of regulatory pathways controlling sodium channel function. In most cases, research focuses on one or a few particular signaling pathways exploring the individual steps between a given stimulus and the target protein, in our case, the sodium channel. This is the way to explore things, dissecting the network, meticulously identifying and characterizing the individual pieces, then reconstructing the complex system. We believe this is the point where cardiac research is behind the possibilities. Continuing the current research strategy may result in new biological signaling pathways and signaling molecules, but it is time to start putting the pieces of the puzzle together. It is time to start making steps toward understanding how the presently known signaling pathways interact and crosstalk in controlling the cardiac sodium channel and, specifically, the late sodium current. We need new, cross-disciplinary research strategies to combine our descriptive data with the concepts of integrative/system biology and computer modeling. We believe that with joined efforts, we can step to a new level of understanding the function of cardiac late sodium current in health and disease.

## Data Availability

No new data were created or analyzed in this study. Data sharing is not applicable to this article.

## Abbreviations

AP	Action Potential
AF	Atrial fibrillation
ATX-II	Anemone toxin II
CaM	Calmodulin
CaMK	Calmodulin Kinase
DAD	Delayed Afterdepolarization
DCM	Dilated Cardiomyopathy
EAD	Early Afterdepolarization
NCX	Na^+^/Ca^2+^ exchanger
NKA	Na^+^/K^+^-ATPase
SGK	Serum- and Glucocorticoid-Inducible Kinase
SSI	Steady State Inactivation
TTX	Tetrodotoxin
VGSC	Voltage Gated Sodium Channel

## References

[1] Horvath B., Banyasz T., Jian Z., Hegyi B., Kistamas K., Nanasi P.P., Izu L.T., Chen-Izu Y. (2013). Dynamics of the late Na^+^ current during cardiac action potential and its contribution to afterdepolarizations. J. Mol. Cell. Cardiol..

[2] Hegyi B., Banyasz T., Izu L.T., Belardinelli L., Bers D.M., Chen-Izu Y. (2018). beta-adrenergic regulation of late Na^+^ current during cardiac action potential is mediated by both PKA and CaMKII. J. Mol. Cell. Cardiol..

[3] Horvath B., Hezso T., Szentandrassy N., Kistamas K., Arpadffy-Lovas T., Varga R., Gazdag P., Veress R., Dienes C., Baranyai D. (2020). Late sodium current in human, canine and guinea pig ventricular myocardium. J. Mol. Cell. Cardiol..

[4] Bengel P., Ahmad S., Tirilomis P., Trum M., Dybkova N., Wagner S., Maier L.S., Hasenfuß G., Sossalla S. (2020). Contribution of the neuronal sodium channel Na(V)1.8 to sodium- and calcium-dependent cellular proarrhythmia. J. Mol. Cell. Cardiol..

[5] Song Y., Belardinelli L. (2018). Enhanced basal late sodium current appears to underlie the age-related prolongation of action potential duration in guinea pig ventricular myocytes. J. Appl. Physiol..

[6] Undrovinas N.A., Maltsev V., Belardinelli L., Sabbah H.N., Undrovinas A. (2010). Late sodium current contributes to diastolic cell Ca^2+^ accumulation in chronic heart failure. J. Physiol. Sci..

[7] Pabel S., Ahmad S., Tirilomis P., Stehle T., Mustroph J., Knierim M., Dybkova N., Bengel P., Holzamer A., Hilker M. (2020). Inhibition of Na(V)1.8 prevents atrial arrhythmogenesis in human and mice. Basic Res. Cardiol..

[8] Wasserstrom J.A., Sharma R., O’Toole M.J., Zheng J., Kelly J.E., Shryock J., Belardinelli L., Aistrup G.L. (2009). Ranolazine Antagonizes the Effects of Increased Late Sodium Current on Intracellular Calcium Cycling in Rat Isolated Intact Heart. J. Pharmacol. Exp. Ther..

[9] Undrovinas A.I., Belardinelli L., Undrovinas N.A., Sabbah H.N. (2006). Ranolazine Improves Abnormal Repolarization and Contraction in Left Ventricular Myocytes of Dogs with Heart Failure by Inhibiting Late Sodium Current. J. Cardiovasc. Electrophysiol..

[10] Sossalla S., Wagner S., Rasenack E.C., Ruff H., Weber S.L., Schöndube F.A., Tirilomis T., Tenderich G., Hasenfuss G., Belardinelli L. (2008). Ranolazine improves diastolic dysfunction in isolated myocardium from failing human hearts—Role of late sodium current and intracellular ion accumulation. J. Mol. Cell. Cardiol..

[11] Sossalla S., Maurer U., Schotola H., Hartmann N., Didie M., Zimmermann W.H., Jacobshagen C., Wagner S., Maier L.S. (2011). Diastolic dysfunction and arrhythmias caused by overexpression of CaMKIIdelta(C) can be reversed by inhibition of late Na(+) current. Basic Res. Cardiol..

[12] Hoyer K., Song Y., Wang D., Phan D., Balschi J., Ingwall J.S., Belardinelli L., Shryock J.C. (2011). Reducing the Late Sodium Current Improves Cardiac Function during Sodium Pump Inhibition by Ouabain. J. Pharmacol. Exp. Ther..

[13] Guo D., Young L., Wu Y., Belardinelli L., Kowey P.R., Yan G.-X. (2010). Increased late sodium current in left atrial myocytes of rabbits with left ventricular hypertrophy: Its role in the genesis of atrial arrhythmias. Am. J. Physiol. Circ. Physiol..

[14] Sossalla S., Kallmeyer B., Wagner S., Mazur M., Maurer U., Toischer K., Schmitto J., Seipelt R., Schöndube F.A., Hasenfuss G. (2010). Altered Na^+^Currents in Atrial Fibrillation: Effects of Ranolazine on Arrhythmias and Contractility in Human Atrial Myocardium. J. Am. Coll. Cardiol..

[15] Zaza A., Belardinelli L., Shryock J.C. (2008). Pathophysiology and pharmacology of the cardiac “late sodium current”. Pharmacol. Ther..

[16] Shryock J.C., Song Y.J., Rajamani S., Antzelevitch C., Belardinelli L. (2013). The arrhythmogenic consequences of increasing late I-Na in the cardiomyocyte. Cardiovasc. Res..

[17] Belardinelli L., Giles W.R., Rajamani S., Karagueuzian H.S., Shryock J.C. (2015). Cardiac late Na^+^ current: Proarrhythmic effects, roles in long QT syndromes, and pathological relationship to CaMKII and oxidative stress. Heart Rhythm. Off. J. Heart Rhythm. Soc..

[18] Bossu A., Houtman M.J.C., Meijborg V.M.F., Varkevisser R., Beekman H.D.M., Dunnink A., De Bakker J.M.T., Mollova N., Rajamani S., Belardinelli L. (2018). Selective late sodium current inhibitor GS-458967 suppresses Torsades de Pointes by mostly affecting perpetuation but not initiation of the arrhythmia. J. Cereb. Blood Flow Metab..

[19] Sicouri S., Belardinelli L., Antzelevitch C. (2013). Antiarrhythmic effects of the highly selective late sodium channel current blocker GS-458967. Heart Rhythm.

[20] Belardinelli L., Liu G., Smith-Maxwell C., Wang W.-Q., El-Bizri N., Hirakawa R., Karpinski S., Li C.H., Hu L., Li X.-J. (2012). A Novel, Potent, and Selective Inhibitor of Cardiac Late Sodium Current Suppresses Experimental Arrhythmias. J. Pharmacol. Exp. Ther..

[21] Wu L., Shryock J.C., Song Y., Li Y., Antzelevitch C., Belardinelli L. (2004). Antiarrhythmic Effects of Ranolazine in a Guinea Pig in Vitro Model of Long-QT Syndrome. J. Pharmacol. Exp. Ther..

[22] Dhalla A.K., Wang W.Q., Dow J., Shryock J.C., Belardinelli L., Bhandari A., Kloner R.A. (2009). Ranolazine, an antianginal agent, markedly reduces ventricular arrhythmias induced by ischemia and ischemia-reperfusion. Am. J. Physiol.-Heart Circ. Physiol..

[23] Zaza A., Rocchetti M. (2013). The late Na^+^ current-origin and pathophysiological relevance. Cardiovasc. Drugs Ther..

[24] Maier L.S., Sossalla S. (2013). The late Na current as a therapeutic target: Where are we?. J. Mol. Cell. Cardiol..

[25] Kistamás K., Hézső T., Horváth B., Nánási P.P. (2020). Late sodium current and calcium homeostasis in arrhythmogenesis. Channels.

[26] Horváth B., Hézső T., Kiss D., Kistamas K., Magyar J., Nánási P.P., Bányász T. (2020). Late Sodium Current Inhibitors as Potential Antiarrhythmic Agents. Front. Pharmacol..

[27] Banyasz T., Szentandrassy N., Magyar J., Szabo Z., Nanasi P., Chen-Izu Y., Izu L. (2014). An Emerging Antiarrhythmic Target: Late Sodium Current. Curr. Pharm. Des..

[28] Rivaud M.R., Delmar M., Remme C.A. (2020). Heritable arrhythmia syndromes associated with abnormal cardiac sodium channel function: Ionic and non-ionic mechanisms. Cardiovasc. Res..

[29] Jia S.B., Lian J.F., Guo D.L., Xue X.L., Patel C., Yang L., Yuan Z.Y., Ma A.Q., Yan G.X. (2011). Modulation of the late sodium current by ATX-II and ranolazine affects the reverse use-dependence and proarrhythmic liability of I-Kr blockade. Br. J. Pharmacol..

[30] Nuyens D., Stengl M., Dugarmaa S., Rossenbacker T., Compernolle V., Rudy Y., Smits J.F., Flameng W., Clancy C.E., Moons L. (2001). Abrupt rate accelerations or premature beats cause life-threatening arrhythmias in mice with long-QT3 syndrome. Nat. Med..

[31] Stafstrom C.E. (2007). Persistent Sodium Current and Its Role in Epilepsy. Epilepsy Curr..

[32] Wengert E.R., Patel M.K. (2020). The Role of the Persistent Sodium Current in Epilepsy. Epilepsy Curr..

[33] Quignard J.-F., Ryckwaert F., Albat B., Nargeot J., Richard S. (1997). A Novel Tetrodotoxin-Sensitive Na sup + Current in Cultured Human Coronary Myocytes. Circ. Res..

[34] Djamgoz M.B., Onkal R. (2013). Persistent Current Blockers of Voltage-Gated Sodium Channels: A Clinical Opportunity for Controlling Metastatic Disease. Recent Patents Anti-Cancer Drug Discov..

[35] Rizzetto R., Rocchetti M., Sala L., Ronchi C., Villa A., Ferrandi M., Molinari I., Bertuzzi F., Zaza A. (2015). Late sodium current (INaL) in pancreatic β-cells. Pflug. Arch. Eur. J. Physiol..

[36] Frankenhaeuser B. (1962). Instantaneous potassium currents in myelinated nerve fibres ofXenopus laevis. J. Physiol..

[37] Frankenhaeuser B. (1963). A quantitative description of potassium currents in myelinated nerve fibres of Xenopus laevis. J. Physiol..

[38] Frankenhaeuser B. (1962). Potassium permeability in myelinated nerve fibres ofXenopus laevis. J. Physiol..

[39] Frankenhaeuser B. (1962). Delayed currents in myelinated nerve fibres of Xenopus laevis investigated with voltage clamp technique. J. Physiol..

[40] Dubois J.M., Bergman C. (1975). Late Sodium Current in Node of Ranvier. Pflug. Arch. -Eur. J. Physiol..

[41] Coraboeuf E., Deroubaix E., Coulombe A. (1979). Effect of Tetrodotoxin on Action Potentials of the Conductiv System in the Dog Heart. Am. J. Physiol..

[42] Attwell D., Cohen I., Eisner D., Ohba M., Ojeda C. (1979). Steady-state TTX-sensitive (window) sodium current in cardiac Purkinje-fibers. Pflug. Arch.-Eur. J. Physiol..

[43] Kiyosue T., Arita M. (1989). Late sodium current and its contribution to action potential configuration in guinea pig ventricular myocytes. Circ. Res..

[44] Clancy C.E., Tateyama M., Liu H., Wehrens X.H., Kass R.S. (2003). Non-equilibrium gating in cardiac Na^+^ channels: An original mechanism of arrhythmia. Circulation.

[45] Ju Y.K., Saint D.A., Gage P.W. (1996). Hypoxia increases persistent sodium current in rat ventricular myocytes. J. Physiol..

[46] Undrovinas A.I., Fleidervish I.A., Makielski J.C. (1992). Inward sodium current at resting potentials in single cardiac myocytes induced by the ischemic metabolite lysophosphatidylcholine. Circ. Res..

[47] Wu J., Corr P.B. (1994). Palmitoyl carnitine modifies sodium currents and induces transient inward current in ventricular myocytes. Am. J. Physiol. Circ. Physiol..

[48] Pezhouman A., Madahian S., Stepanyan H., Ghukasyan H., Qu Z., Belardinelli L., Karagueuzian H.S. (2013). Selective inhibition of late sodium current suppresses ventricular tachycardia and fibrillation in intact rat hearts. Heart Rhythm.

[49] Soliman D., Wang L., Hamming K.S.C., Yang W., Fatehi M., Carter C.C., Clanachan A.S., Light P.E. (2012). Late Sodium Current Inhibition Alone with Ranolazine Is Sufficient to Reduce Ischemia- and Cardiac Glycoside-Induced Calcium Overload and Contractile Dysfunction Mediated by Reverse-Mode Sodium/Calcium Exchange. J. Pharmacol. Exp. Ther..

[50] Pignier C., Rougier J.-S., Vié B., Culié C., Verscheure Y., Vacher B., Abriel H., Le Grand B. (2010). Selective inhibition of persistent sodium current by F 15845 prevents ischaemia-induced arrhythmias. J. Cereb. Blood Flow Metab..

[51] Antoons G., Oros A., Beekman J.D., Engelen M.A., Houtman M.J., Belardinelli L., Stengl M., Vos M.A. (2010). Late Na^+^ Current Inhibition by Ranolazine Reduces Torsades de Pointes in the Chronic Atrioventricular Block Dog Model. J. Am. Coll. Cardiol..

[52] Belardinelli L., Antzelevitch C., Fraser H. (2004). Inhibition of late (sustained/persistent) sodium current: A potential drug target to reduce intracellular sodium-dependent calcium overload and its detrimental effects on cardiomyocyte function. Eur. Heart J. Suppl..

[53] Rambarat C.A., Elgendy I.Y., Handberg E.M., Merz C.N.B., Wei J., Minissian M.B., Nelson M.D., Thomson L.E.J., Berman D.S., Shaw L.J. (2019). Late sodium channel blockade improves angina and myocardial perfusion in patients with severe coronary microvascular dysfunction: Women’s Ischemia Syndrome Evaluation-Coronary Vascular Dysfunction ancillary study. Int. J. Cardiol..

[54] Ton A.T., Nguyen W., Sweat K., Miron Y., Hernandez E., Wong T., Geft V., Macias A., Espinoza A., Truong K. (2021). Arrhythmogenic and antiarrhythmic actions of late sustained sodium current in the adult human heart. Sci. Rep..

[55] Dong C., Wang Y., Ma A., Wang T. (2020). Life Cycle of the Cardiac Voltage-Gated Sodium Channel NaV1.5. Front. Physiol..

[56] Catterall W.A., Goldin A.L., Waxman S.G. (2005). International Union of Pharmacology. XLVII. Nomenclature and Structure-Function Relationships of Voltage-Gated Sodium Channels. Pharmacol. Rev..

[57] Zimmer T., Haufe V., Blechschmidt S. (2014). Voltage-gated sodium channels in the mammalian heart. Glob. Cardiol. Sci. Pract..

[58] Godazgar M., Zhang Q., Chibalina M.V., Rorsman P. (2018). Biphasic voltage-dependent inactivation of human Na(V)1.3, 1.6 and 1.7 Na^+^ channels expressed in rodent insulin-secreting cells. J. Physiol.-Lond..

[59] Maltsev V.A., Undrovinas A. (2008). Late sodium current in failing heart: Friend or foe?. Prog. Biophys. Mol. Biol..

[60] Pereon Y., Lande G., Demolombe S., Tich S.N.T., Sternberg D., Le Marec H., David A. (2003). Paramyotonia congenita with an SCN4A mutation affecting cardiac repolarization. Neurology.

[61] Biet M., Barajas-Martínez H., Ton A.-T., Delabre J.-F., Morin N., Dumaine R. (2012). About half of the late sodium current in cardiac myocytes from dog ventricle is due to non-cardiac-type Na^+^ channels. J. Mol. Cell. Cardiol..

[62] Yang T., Atack T.C., Stroud D.M., Zhang W., Hall L., Roden D.M. (2012). Blocking Scn10a channels in heart reduces late sodium current and is antiarrhythmic. Circ. Res..

[63] Haufe V., Cordeiro J.M., Zimmer T., Wu Y.S., Schiccitano S., Benndorf K., Dumaine R. (2005). Contribution of neuronal sodium channels to the cardiac fast sodium current INa is greater in dog heart Purkinje fibers than in ventricles. Cardiovasc. Res..

[64] Maier S.K.G., Westenbroek R.E., Schenkman K.A., Feigl E.O., Scheuer T., Catterall W.A. (2002). An unexpected role for brain-type sodium channels in coupling of cell surface depolarization to contraction in the heart. Proc. Natl. Acad. Sci. USA.

[65] Westenbroek R.E., Bischoff S., Fu Y., Maier S.K., Catterall W.A., Scheuer T. (2013). Localization of sodium channel subtypes in mouse ventricular myocytes using quantitative immunocytochemistry. J. Mol. Cell. Cardiol..

[66] Haufe V., Camacho J.A., Dumaine R., Günther B., Bollensdorff C., Von Banchet G.S., Benndorf K., Zimmer T. (2005). Expression pattern of neuronal and skeletal muscle voltage-gated Na^+^channels in the developing mouse heart. J. Physiol..

[67] Blechschmidt S., Haufe V., Benndorf K., Zimmer T. (2008). Voltage-gated Na^+^ channel transcript patterns in the mammalian heart are species-dependent. Prog. Biophys. Mol. Biol..

[68] Chambers J.C., Zhao J., Terracciano C.M.N., Bezzina C.R., Zhang W., Kaba R., Navaratnarajah M., Lotlikar A., Sehmi J.S., Kooner M.K. (2010). Genetic variation in SCN10A influences cardiac conduction. Nat. Genet..

[69] Verkerk A.O., Remme C.A., Schumacher C.A., Scicluna B.P., Wolswinkel R., de Jonge B., Bezzina C.R., Veldkamp M.W. (2012). Functional Nav1.8 channels in intracardiac neurons: The link between SCN10A and cardiac electrophysiology. Circ. Res..

[70] Jabbari J., Olesen M.S., Yuan L., Nielsen J.B., Liang B., Macri V., Christophersen I.E., Nielsen N., Sajadieh A., Ellinor P.T. (2015). Common and rare variants in SCN10A modulate the risk of atrial fibrillation. Circ. Cardiovasc. Genet..

[71] Savio-Galimberti E., Weeke P., Muhammad R., Blair M., Ansari S., Short L., Atack T.C., Kor K., Vanoye C.G., Olesen M.S. (2014). SCN10A/Nav1.8 modulation of peak and late sodium currents in patients with early onset atrial fibrillation. Cardiovasc. Res..

[72] Stroud D.M., Yang T., Bersell K., Kryshtal D.O., Nagao S., Shaffer C., Short L., Hall L., Atack T.C., Zhang W. (2016). Contrasting Nav1.8 Activity in *Scn10a^−/−^* Ventricular Myocytes and the Intact Heart. J. Am. Heart Assoc..

[73] Macri V., Brody J.A., Arking D.E., Hucker W.J., Yin X., Lin H., Mills R.W., Sinner M.F., Lubitz S.A., Liu C.-T. (2018). Common Coding Variants in SCN10A Are Associated with the Nav1.8 Late Current and Cardiac Conduction. Circ. Genom. Precis. Med..

[74] Maier L.S., Sossalla S., Schulze-Bahr E. (2018). SCN10A-Dependent Late I(Na) Current: Never Too Late for Cardiac Conduction?. Circ. Genom. Precis. Med..

[75] Li N., Kalyanasundaram A., Hansen B.J., Artiga E.J., Sharma R., Abudulwahed S.H., Helfrich K.M., Rozenberg G., Wu P.-J., Zakharkin S. (2020). Impaired neuronal sodium channels cause intranodal conduction failure and reentrant arrhythmias in human sinoatrial node. Nat. Commun..

[76] Casini S., Marchal G.A., Kawasaki M., Nariswari F.A., Portero V., van den Berg N.W.E., Guan K., Driessen A.H.G., Veldkamp M.W., Mengarelli I. (2019). Absence of Functional Na(v)1.8 Channels in Non-diseased Atrial and Ventricular Cardiomyocytes. Cardiovasc. Drugs Ther..

[77] Facer P., Punjabi P.P., Abrari A., Kaba R.A., Severs N.J., Chambers J., Kooner J.S., Anand P. (2011). Localisation of SCN10A Gene Product Nav1.8 and Novel Pain-Related Ion Channels in Human Heart. Int. Heart J..

[78] Xi Y., Wu G., Yang L., Han K., Du Y., Wang T., Lei X., Bai X., Ma A. (2009). Increased late sodium currents are related to transcription of neuronal isoforms in a pressure-overload model. Eur. J. Heart Fail..

[79] Ahmad S., Tirilomis P., Pabel S., Dybkova N., Hartmann N., Molina C.E., Tirilomis T., Kutschka I., Frey N., Maier L.S. (2019). The functional consequences of sodium channel Na(V)1.8 in human left ventricular hypertrophy. Esc. Heart Fail..

[80] Dybkova N., Ahmad S., Pabel S., Tirilomis P., Hartmann N., Fischer T.H., Bengel P., Tirilomis T., Ljubojevic S., Renner A. (2018). Differential regulation of sodium channels as a novel proarrhythmic mechanism in the human failing heart. Cardiovasc. Res..

[81] Brackenbury W.J., Isom L.L. (2011). Na Channel β Subunits: Overachievers of the Ion Channel Family. Front. Pharmacol..

[82] Cusdin F.S., Clare J.J., Jackson A.P. (2008). Trafficking and Cellular Distribution of Voltage-Gated Sodium Channels. Traffic.

[83] Kazen-Gillespie K.A., Ragsdale D.S., D’Andrea M.R., Mattei L.N., Rogers K.E., Isom L.L. (2000). Cloning, localization, and functional expression of sodium channel beta1A subunits. J. Biol. Chem..

[84] Qin N., D’Andrea M.R., Lubin M.-L., Shafaee N., Codd E., Correa A.M. (2003). Molecular cloning and functional expression of the human sodium channel beta1B subunit, a novel splicing variant of the beta1 subunit. JBIC J. Biol. Inorg. Chem..

[85] Llongueras J.P., Das S., De Waele J., Capulzini L., Sorgente A., Van Petegem F., Bosmans F. (2020). Biophysical Investigation of Sodium Channel Interaction with β-Subunit Variants Associated with Arrhythmias. Bioelectricity.

[86] Isaac E., Cooper S.M., Jones S.A., Loubani M. (2020). Do age-associated changes of voltage-gated sodium channel isoforms expressed in the mammalian heart predispose the elderly to atrial fibrillation?. World J. Cardiol..

[87] Maltsev V.A., Kyle J.W., Undrovinas A. (2009). Late Na(+) current produced by human cardiac Na(+) channel isoform Na(v)1.5 is modulated by its beta(1) subunit. J. Physiol. Sci..

[88] Bouza A.A., Isom L.L. (2017). Voltage-Gated Sodium Channel β Subunits and Their Related Diseases. Volt.-Gated Sodium Channels.

[89] Angsutararux P., Zhu W., Voelker T.L., Silva J.R. (2021). Molecular Pathology of Sodium Channel Beta-Subunit Variants. Front. Pharmacol..

[90] Grieco T.M., Malhotra J.D., Chen C., Isom L.L., Raman I.M. (2005). Open-channel block by the cytoplasmic tail of sodium channel beta4 as a mechanism for resurgent sodium current. Neuron.

[91] Clatot J., Hoshi M., Wan X., Liu H., Jain A., Shinlapawittayatorn K., Marionneau C., Ficker E., Eckhard F., Deschênes I. (2017). Voltage-gated sodium channels assemble and gate as dimers. Nat. Commun..

[92] Clatot J., Zheng Y., Girardeau A., Liu H., Laurita K.R., Marionneau C., Deschênes I. (2018). Mutant voltage-gated Na^+^ channels can exert a dominant negative effect through coupled gating. Am. J. Physiol. Circ. Physiol..

[93] Rühlmann A.H., Körner J., Hausmann R., Bebrivenski N., Neuhof C., Detro-Dassen S., Hautvast P., Benasolo C.A., Meents J., Machtens J.-P. (2020). Uncoupling sodium channel dimers restores the phenotype of a pain-linked Nav1.7 channel mutation. Br. J. Pharmacol..

[94] Eshed-Eisenbach Y., Peles E. (2019). The clustering of voltage-gated sodium channels in various excitable membranes. Dev. Neurobiol..

[95] Salvage S.C., Rees J.S., McStea A., Hirsch M., Wang L., Tynan C.J., Reed M.W., Irons J.R., Butler R., Thompson A.J. (2020). Supramolecular clustering of the cardiac sodium channel Nav1.5 in HEK293F cells, with and without the auxiliary beta 3-subunit. Faseb J..

[96] Veeraraghavan R., Radwański P.B. (2018). Sodium channel clusters: Harmonizing the cardiac conduction orchestra. J. Physiol..

[97] Hichri E., Abriel H., Kucera J.P. (2018). Distribution of cardiac sodium channels in clusters potentiates ephaptic interactions in the intercalated disc. J. Physiol..

[98] Bhargava A., Lin X., Novak P., Mehta K., Korchev Y., Delmar M., Gorelik J. (2013). Super-resolution Scanning Patch Clamp Reveals Clustering of Functional Ion Channels in Adult Ventricular Myocyte. Circ. Res..

[99] Lin X., Liu N., Lu J., Zhang J., Anumonwo J.M., Isom L., Fishman G.I., Delmar M. (2011). Subcellular heterogeneity of sodium current properties in adult cardiac ventricular myocytes. Heart Rhythm.

[100] Salvage S.C., Huang C.L., Jackson A.P. (2020). Cell-Adhesion Properties of β-Subunits in the Regulation of Cardiomyocyte Sodium Channel. Biomolecules.

[101] Verkerk A.O., van Ginneken A.C., van Veen T.A., Tan H.L. (2007). Effects of heart failure on brain-type Na^+^ channels in rabbit ventricular myocytes. Europace.

[102] Struckman H.L., Baine S., Thomas J., Mezache L., Mykytyn K., Györke S., Radwański P.B., Veeraraghavan R. (2020). Super-Resolution Imaging Using a Novel High-Fidelity Antibody Reveals Close Association of the Neuronal Sodium Channel Na(V)1.6 with Ryanodine Receptors in Cardiac Muscle, Microscopy and microanalysis: The official journal of Microscopy Society of America, Microbeam Analysis Society. Microsc. Soc. Can..

[103] Munger M.A., Olğar Y., Koleske M.L., Struckman H.L., Mandrioli J., Lou Q., Bonila I., Kim K., Mondragon R.R., Priori S.G. (2020). Tetrodotoxin-Sensitive Neuronal-Type Na + Channels: A Novel and Druggable Target for Prevention of Atrial Fibrillation. J. Am. Heart Assoc..

[104] Jayasinghe I., Clowsley A.H., De Langen O., Sali S.S., Crossman D., Soeller C. (2018). Shining New Light on the Structural Determinants of Cardiac Couplon Function: Insights from Ten Years of Nanoscale Microscopy. Front. Physiol..

[105] Song Y., Belardinelli L. (2017). Basal late sodium current is a significant contributor to the duration of action potential of guinea pig ventricular myocytes. Physiol. Rep..

[106] Sheu S.S., Lederer W.J. (1985). Lidocaine’s negative inotropic and antiarrhythmic actions. Dependence on shortening of action potential duration and reduction of intracellular sodium activity. Circ. Res..

[107] Makielski J.C., Farley A.L. (2006). Na^+^ Current in Human Ventricle: Implications for Sodium Loading and Homeostasis. J. Cardiovasc. Electrophysiol..

[108] Hegyi B., Bányász T., Shannon T.R., Chen-Izu Y., Izu L.T. (2016). Electrophysiological Determination of Submembrane Na^+^ Concentration in Cardiac Myocytes. Biophys. J..

[109] Despa S. (2018). Myocyte [Na^+^]i Dysregulation in Heart Failure and Diabetic Cardiomyopathy. Front. Physiol..

[110] Despa S., Bers D. (2013). Na^+^ transport in the normal and failing heart—Remember the balance. J. Mol. Cell. Cardiol..

[111] Schramm M., Klieber H.G., Daut J. (1994). The energy-expenditure of actomyosin-ATPase, Ca^2+^-ATPase and Na^+^/K+-ATPase in guinea-pig cardiac ventricular muscle. J. Physiol.-Lond..

[112] Brill D.M., Wasserstrom J.A. (1986). Intracellular sodium and the positive inotropic effect of veratridine and cardiac glycoside in sheep Purkinje fibers. Circ. Res..

[113] Zhang T., Brown J.H. (2004). Role of Ca^2+^/calmodulin-dependent protein kinase II in cardiac hypertrophy and heart failure. Cardiovasc. Res..

[114] Anderson M.E. (2005). Calmodulin kinase signaling in heart: An intriguing candidate target for therapy of myocardial dysfunction and arrhythmias. Pharmacol. Ther..

[115] Bers D.M., Grandi E. (2009). Calcium/Calmodulin-dependent Kinase II Regulation of Cardiac Ion Channels. J. Cardiovasc. Pharmacol..

[116] Bers D.M. (2002). Cardiac excitation-contraction coupling. Nature.

[117] Zhu W., Woo A.Y., Yang D., Cheng H., Crow M.T., Xiao R.P. (2007). Activation of CaMKIIdeltaC is a common intermediate of diverse death stimuli-induced heart muscle cell apoptosis. J. Biol. Chem..

[118] Chen X., Zhang X., Kubo H., Harris D.M., Mills G.D., Moyer J., Berretta R., Potts S.T., Marsh J.D., Houser S.R. (2005). Ca^2+^ Influx–Induced Sarcoplasmic Reticulum Ca^2+^ Overload Causes Mitochondrial-Dependent Apoptosis in Ventricular Myocytes. Circ. Res..

[119] Petroff M.V., Salas M.A., Said M., Valverde C.A., Sapia L., Portiansky E., Hajjar R.J., Kranias E.G., Mundiña-Weilenmann C., Mattiazzi A. (2007). CaMKII inhibition protects against necrosis and apoptosis in irreversible ischemia–reperfusion injury. Cardiovasc. Res..

[120] Noble D., Noble P.J. (2006). Late sodium current in the pathophysiology of cardiovascular disease: Consequences of sodium-calcium overload. Heart.

[121] Zhang S., Ma J.-H., Zhang P.-H., Luo A.-T., Ren Z.-Q., Kong L.-H. (2012). Sophocarpine Attenuates the Na^+^-dependent Ca^2+^ Overload Induced by Anemonia Sulcata Toxin—Increased Late Sodium Current in Rabbit Ventricular Myocytes. J. Cardiovasc. Pharmacol..

[122] Philippaert K., Kalyaanamoorthy S., Fatehi M., Long W., Soni S., Byrne N.J., Barr A., Singh J., Wong J., Palechuk T. (2021). Cardiac Late Sodium Channel Current Is a Molecular Target for the Sodium/Glucose Cotransporter 2 Inhibitor Empagliflozin. Circulation.

[123] Bengel P., Dybkova N., Tirilomis P., Ahmad S., Hartmann N., Mohamed B.A., Krekeler M.C., Maurer W., Pabel S., Trum M. (2021). Detrimental proarrhythmogenic interaction of Ca^2+^/calmodulin-dependent protein kinase II and Na(V)1.8 in heart failure. Nat. Commun..

[124] Hilgemann D.W. (2019). Control of cardiac contraction by sodium: Promises, reckonings, and new beginnings. Cell Calcium.

[125] Aksentijevic D., O’Brien B.A., Eykyn T.R., Shattock M. (2018). Is there a causal link between intracellular Na elevation and metabolic remodelling in cardiac hypertrophy?. Biochem. Soc. Trans..

[126] Phuket T., Covarrubias M. (2009). Kv4 Channels Underlie the Subthreshold-Operating A-type K+-current in Nociceptive Dorsal Root Ganglion Neurons. Front. Mol. Neurosci..

[127] Antoons G., Volders P.G.A., Stankovicova T., Bito V., Stengl M., Vos M.A., Sipido K.R. (2007). Window Ca^2+^current and its modulation by Ca^2+^release in hypertrophied cardiac myocytes from dogs with chronic atrioventricular block. J. Physiol..

[128] Morita N., Lee J.H., Xie Y., Sovari A., Qu Z., Weiss J.N., Karagueuzian H.S. (2011). Suppression of Re-Entrant and Multifocal Ventricular Fibrillation by the Late Sodium Current Blocker Ranolazine. J. Am. Coll. Cardiol..

[129] Liu H., Sun H.-Y., Lau C.-P., Li G.-R. (2007). Regulation of voltage-gated cardiac sodium current by epidermal growth factor receptor kinase in guinea pig ventricular myocytes. J. Mol. Cell. Cardiol..

[130] Wang D.W., Yazawa K., George A.L., Bennett P.B. (1996). Characterization of human cardiac Na^+^ channel mutations in the congenital long QT syndrome. Proc. Natl. Acad. Sci. USA.

[131] Moreno J.D., Clancy C.E. (2011). Pathophysiology of the cardiac late Na current and its potential as a drug target. J. Mol. Cell. Cardiol..

[132] Beyder A., Rae J.L., Bernard C., Strege P.R., Sachs F., Farrugia G. (2010). Mechanosensitivity of Na(v)1.5, a voltage-sensitive sodium channel. J. Physiol.-Lond..

[133] O’Reilly-Shah V., Wingo T.L., Weiss K.L., Williams C.K., Balser J.R., Chazin W.J. (2006). Calcium-dependent regulation of the voltage-gated sodium channel hH1: Intrinsic and extrinsic sensors use a common molecular switch. Proc. Natl. Acad. Sci. USA.

[134] Wingo T.L., Shah V.N., Anderson M.E., Lybrand T.P., Chazin W.J., Balser J.R. (2004). An EF-hand in the sodium channel couples intracellular calcium to cardiac excitability. Nat. Struct. Mol. Biol..

[135] Ruan Y., Liu N., Priori S.G. (2009). Sodium channel mutations and arrhythmias. Nat. Rev. Cardiol..

[136] Rivaud M.R., Baartscheer A., Verkerk A.O., Beekman L., Rajamani S., Belardinelli L., Bezzina C.R., Remme C.A. (2018). Enhanced late sodium current underlies pro-arrhythmic intracellular sodium and calcium dysregulation in murine sodium channelopathy. Int. J. Cardiol..

[137] Rivaud M.R., Marchal G.A., Wolswinkel R., Jansen J.A., van der Made I., Beekman L., Ruiz-Villalba A., Baartscheer A., Rajamani S., Belardinelli L. (2020). Functional modulation of atrio-ventricular conduction by enhanced late sodium current and calcium-dependent mechanisms in Scn5a(1798insDl+) mice. Europace.

[138] Peters C.H., Watkins A.R., Poirier O.L., Ruben P.C. (2020). E1784K, the most common Brugada syndrome and long-QT syndrome type 3 mutant, disrupts sodium channel inactivation through two separate mechanisms. J. Gen. Physiol..

[139] Mitsuiye T., Noma A. (2002). Inactivation of Cardiac Na^+^ Channel Simply through Open States as Revealed by Single-Channel Analysis in Guinea Pig Ventricular Myocytes. Jpn. J. Physiol..

[140] Maltsev V., Undrovinas A.I. (2006). A multi-modal composition of the late Na^+^ current in human ventricular cardiomyocytes. Cardiovasc. Res..

[141] Scanley B.E., Hanck D.A., Chay T., Fozzard H.A. (1990). Kinetic-analysis of single sodium-channels from canine cardiac Purkinje-cells. J. Gen. Physiol..

[142] Patlak J.B., Ortiz M. (1989). Kinetic diversity of Na^+^ channel bursts in frog skeletal muscle. J. Gen. Physiol..

[143] Patlak J.B., Ortiz M. (1986). Two modes of gating during late Na^+^ channel currents in frog sartorius muscle. J. Gen. Physiol..

[144] Patlak J.B., Ortiz M. (1985). Slow currents through single sodium channels of the adult rat heart. J. Gen. Physiol..

[145] Kohlhardt M., Frobe U., Herzig J.W. (1987). Properties of normal and noninactivating single cardic Na^+^ channels. Proc. R. Soc. Ser. B-Biol. Sci..

[146] Bezzina C., Veldkamp M.W., Berg M.V.D., Postma A., Rook M.B., Viersma J.-W., van Langen I.M., Tan-Sindhunata G., Bink-Boelkens M.T.E., van der Hout A.H. (1999). A Single Na + Channel Mutation Causing Both Long-QT and Brugada Syndromes. Circ. Res..

[147] Maltsev V., Silverman N., Sabbah H.N., Undrovinas A.I. (2007). Chronic heart failure slows late sodium current in human and canine ventricular myocytes: Implications for repolarization variability. Eur. J. Heart Fail..

[148] Valdivia C.R., Chu W.W., Pu J., Foell J.D., Haworth R.A., Wolff M.R., Kamp T., Makielski J.C. (2005). Increased late sodium current in myocytes from a canine heart failure model and from failing human heart. J. Mol. Cell. Cardiol..

[149] Song Y., Shryock J.C., Belardinelli L. (2008). An increase of late sodium current induces delayed afterdepolarizations and sustained triggered activity in atrial myocytes. Am. J. Physiol. Circ. Physiol..

[150] Trenor B., Cardona K., Gómez J.F., Rajamani S., Jr J.M.F., Belardinelli L., Saiz J. (2012). Simulation and Mechanistic Investigation of the Arrhythmogenic Role of the Late Sodium Current in Human Heart Failure. PLoS ONE.

[151] Wu L., Shryock J.C., Song Y., Belardinelli L. (2005). An Increase in Late Sodium Current Potentiates the Proarrhythmic Activities of Low-Risk QT-Prolonging Drugs in Female Rabbit Hearts. J. Pharmacol. Exp. Ther..

[152] Belardinelli L., Shryock J.C., Fraser H. (2006). Inhibition of the late sodium current as a potential cardioprotective principle: Effects of the late sodium current inhibitor ranolazine. Heart.

[153] Magyar J., Kiper C.E., Dumaine R., Burgess D.E., Bányász T., Satin J. (2004). Divergent action potential morphologies reveal nonequilibrium properties of human cardiac Na channels. Cardiovasc. Res..

[154] Bant J.S., Raman I.M. (2010). Control of transient, resurgent, and persistent current by open-channel block by Na channel β4 in cultured cerebellar granule neurons. Proc. Natl. Acad. Sci. USA.

[155] Grieco T.M., Raman I.M. (2004). Production of Resurgent Current in Na_V_1.6-Null Purkinje Neurons by Slowing Sodium Channel Inactivation with β-Pompilidotoxin. J. Neurosci..

[156] Raman I.M., Bean B.P. (1997). Resurgent sodium current and action potential formation in dissociated cerebellar Purkinje neurons. J. Neurosci..

[157] Eijkelkamp N., Linley J., Baker M.D., Minett M.S., Cregg R., Werdehausen R., Rugiero F., Wood J.N. (2012). Neurological perspectives on voltage-gated sodium channels. Brain.

[158] Schiavon E., Sacco T., Cassulini R.R., Gurrola G., Tempia F., Possani L.D., Wanke E. (2006). Resurgent current and voltage sensor trapping enhanced activation by a beta-scorpion toxin solely in Nav1.6 channel. Significance in mice Purkinje neurons. J. Biol. Chem..

[159] DeMarco K.R., Clancy C.E., French R.J., Noskov S.Y. (2016). Cardiac Na Channels: Structure to Function. Na Channels from Phyla to Function.

[160] Maier L.S. (2011). CaMKII regulation of voltage-gated sodium channels and cell excitability. Heart Rhythm. Off. J. Heart Rhythm. Soc..

[161] Maltsev V.A., Reznikov V., Undrovinas N.A., Sabbah H.N., Undrovinas A. (2008). Modulation of late sodium current by Ca^2+^, calmodulin, and CaMKII in normal and failing dog cardiomyocytes: Similarities and differences. Am. J. Physiol. Circ. Physiol..

[162] Lewit-Bentley A., Réty S. (2001). EF-hand calcium-binding proteins. Curr. Opin. Struct. Biol..

[163] Van Petegem F., Lobo P.A., Ahern C.A. (2012). Seeing the Forest through the Trees: Towards a Unified View on Physiological Calcium Regulation of Voltage-Gated Sodium Channels. Biophys. J..

[164] Tan H.L., Kupershmidt S., Zhang R., Stepanovic S., Roden D.M., Wilde A.A.M., Anderson M.E., Balser J.R. (2002). A calcium sensor in the sodium channel modulates cardiac excitability. Nature.

[165] Kim J., Ghosh S., Liu H., Tateyama M., Kass R.S., Pitt G.S. (2004). Calmodulin mediates Ca^2+^ sensitivity of sodium channels. J. Biol. Chem..

[166] Biswas S., DiSilvestre D., Tian Y., Halperin V.L., Tomaselli G.F. (2009). Calcium-Mediated Dual-Mode Regulation of Cardiac Sodium Channel Gating. Circ. Res..

[167] Sarhan M.F., Tung C.C., van Petegem F., Ahern C.A. (2012). Crystallographic basis for calcium regulation of sodium channels. Proc. Natl. Acad. Sci. USA.

[168] Ulbricht W. (2005). Sodium Channel Inactivation: Molecular Determinants and Modulation. Physiol. Rev..

[169] Murray K.T., Hu N., Daw J.R., Shin H.-G., Watson M.T., Mashburn A.B., George A.L. (1997). Functional Effects of Protein Kinase C Activation on the Human Cardiac Na sup + Channel. Circ. Res..

[170] Scheuer T. (2011). Regulation of sodium channel activity by phosphorylation. Semin. Cell Dev. Biol..

[171] Qu Y., Rogers J.C., Tanada T.N., Catterall W.A., Scheuer T. (1996). Phosphorylation of S1505 in the cardiac Na^+^ channel inactivation gate is required for modulation by protein kinase C. J. Gen. Physiol..

[172] Wang Z.Z., Vermij S.H., Sottas V., Shestak A., Ross-Kaschitza D., Zaklyazminskaya E.V., Hudmon A., Pitt G.S., Rougier J.S., Abriel H. (2020). Calmodulin binds to the N-terminal domain of the cardiac sodium channel Na(v)1.5. Channels.

[173] Hudmon A., Schulman H. (2002). Structure-function of the multifunctional Ca^2+^/calmodulin-dependent protein kinase II. Biochem. J..

[174] Marionneau C., Lichti C.F., Lindenbaum P., Charpentier F., Nerbonne J.M., Townsend R.R., Mérot J. (2012). Mass Spectrometry-Based Identification of Native Cardiac Nav1.5 Channel α Subunit Phosphorylation Sites. J. Proteome Res..

[175] Murphy B.J., Rogers J., Perdichizzi A.P., Colvin A.A., Catterall W.A. (1996). cAMP-dependent phosphorylation of two sites in the alpha subunit of the cardiac sodium channel. J. Biol. Chem..

[176] Rook M.B., Evers M.M., Vos M.A., Bierhuizen M.F. (2012). Biology of cardiac sodium channel Nav1.5 expression. Cardiovasc. Res..

[177] Zhou J., Yi J., Hu N., George A.L., Murray K.T. (2000). Activation of Protein Kinase A Modulates Trafficking of the Human Cardiac Sodium Channel in Xenopus Oocytes. Circ. Res..

[178] Wagner S., Dybkova N., Rasenack E.C.L., Jacobshagen C., Fabritz L., Kirchhof P., Maier S.K.G., Zhang T., Hasenfuss G., Brown J.H. (2006). Ca^2+^/calmodulin-dependent protein kinase II regulates cardiac Na^+^ channels. J. Clin. Investig..

[179] Ashpole N.M., Herren A.W., Ginsburg K.S., Brogan J.D., Johnson D.E., Cummins T.R., Bers D.M., Hudmon A. (2012). Ca^2+^/Calmodulin-dependent Protein Kinase II (CaMKII) Regulates Cardiac Sodium Channel NaV1.5 Gating by Multiple Phosphorylation Sites. J. Biol. Chem..

[180] Koval O.M., Snyder J.S., Wolf R.M., Pavlovicz R.E., Glynn P., Curran J., Leymaster N.D., Dun W., Wright P.J., Cardona N. (2012). Ca^2+^/calmodulin-dependent protein kinase II-based regulation of voltage-gated Na^+^ channel in cardiac disease. Circulation.

[181] Aiba T., Hesketh G.G., Liu T., Carlisle R., Villa-Abrille M.C., O’Rourke B., Akar F.G., Tomaselli G.F. (2010). Na^+^ channel regulation by Ca^2+^/calmodulin and Ca^2+^/calmodulin-dependent protein kinase II in guinea-pig ventricular myocytes. Cardiovasc. Res..

[182] Fouda M.A., Ruben P.C. (2021). Protein Kinases Mediate Anti-Inflammatory Effects of Cannabidiol and Estradiol Against High Glucose in Cardiac Sodium Channels. Front. Pharmacol..

[183] Palaniyandi S.S., Sun L., Ferreira J.C.B., Mochly-Rosen D. (2008). Protein kinase C in heart failure: A therapeutic target?. Cardiovasc. Res..

[184] Mellor H., Parker P. (1998). The extended protein kinase C superfamily. Biochem. J..

[185] Herren A.W., Bers D.M., Grandi E. (2013). Post-translational modifications of the cardiac Na channel: Contribution of CaMKII-dependent phosphorylation to acquired arrhythmias. Am. J. Physiol.-Heart Circ. Physiol..

[186] Grimm M., Ling H.Y., Brown J.H. (2011). Crossing signals: Relationships between beta-adrenergic stimulation and CaMKII activation. Heart Rhythm. Off. J. Heart Rhythm. Soc..

[187] Tateyama M., Rivolta I., Clancy C.E., Kass R.S. (2003). Modulation of Cardiac Sodium Channel Gating by Protein Kinase A Can Be Altered by Disease-linked Mutation. J. Biol. Chem..

[188] Ma J., Luo A., Wu L., Wan W., Zhang P., Ren Z., Zhang S., Qian C., Shryock J.C., Belardinelli L. (2012). Calmodulin kinase II and protein kinase C mediate the effect of increased intracellular calcium to augment late sodium current in rabbit ventricular myocytes. Am. J. Physiol. Physiol..

[189] Qu Y., Rogers J., Tanada T., Scheuer T., Catterall W.A. (1994). Modulation of cardiac Na^+^ channels expressed in a mammalian cell line and in ventricular myocytes by protein kinase C. Proc. Natl. Acad. Sci. USA.

[190] Aoyama T., Matsui T., Novikov M., Park J., Hemmings B., Rosenzweig A. (2005). Serum and Glucocorticoid-Responsive Kinase-1 Regulates Cardiomyocyte Survival and Hypertrophic Response. Circulation.

[191] Lang F., Shumilina E. (2012). Regulation of ion channels by the serum- and glucocorticoid-inducible kinase SGK1. FASEB J..

[192] Lang F., Böhmer C., Palmada M., Seebohm G., Strutz-Seebohm N., Vallon V. (2006). (Patho)physiological Significance of the Serum- and Glucocorticoid-Inducible Kinase Isoforms. Physiol. Rev..

[193] Kobayashi T., Deak M., Morrice N., Cohen P. (1999). Characterization of the structure and regulation of two novel isoforms of serum- and glucocorticoid-induced protein kinase. Biochem. J..

[194] Murphy L., Renodin D., Antzelevitch C., Di Diego J.M., Cordeiro J.M. (2011). Extracellular proton depression of peak and late Na^+^ current in the canine left ventricle. Am. J. Physiol. Circ. Physiol..

[195] Murphy L., Renodin D.M., Antzelevitch C., Di Diego J.M., Cordeiro J.M. (2011). Extracellular Proton Modulation of Peak and Late Sodium Current in the Canine Left Ventricle. Biophys. J..

[196] Jones D., Peters C.H., Allard C.R., Claydon T., Ruben P.C. (2013). Proton Sensors in the Pore Domain of the Cardiac Voltage-gated Sodium Channel. J. Biol. Chem..

[197] Das S., Aiba T., Rosenberg M., Hessler K., Xiao C., Quintero P.A., Ottaviano F.G., Knight A.C., Graham E.L., Boström P. (2012). Pathological Role of Serum- and Glucocorticoid-Regulated Kinase 1 in Adverse Ventricular Remodeling. Circulation.

[198] Boehmer C., Wilhelm V., Palmada M., Wallisch S., Henke G., Brinkmeier H., Cohen P., Pieske B., Lang F. (2003). Serum and glucocorticoid inducible kinases in the regulation of the cardiac sodium channel SCN5A. Cardiovasc. Res..

[199] Fahmi A., Forhead A., Fowden A., Vandenberg J., Forhead A., Vandenberg J. (2004). Cortisol influences the ontogeny of both alpha- and beta-subunits of the cardiac sodium channel in fetal sheep. J. Endocrinol..

[200] Nguyenthi A., Ruizceretti E., Schanne O.F. (1981). Electrophysiologic effects and electrolite changes in total myocardial ischemia. Can. J. Physiol. Pharmacol..

[201] Jones D., Peters C., Tolhurst S., Claydon T., Ruben P. (2011). Extracellular Proton Modulation of the Cardiac Voltage-Gated Sodium Channel, NaV1.5. Biophys. J..

[202] Jones D., Claydon T., Ruben P. (2013). Extracellular Protons Inhibit Charge Immobilization in the Cardiac Voltage-Gated Sodium Channel. Biophys. J..

[203] Tang Q., Ma J.H., Zhang P.H., Wan W., Kong L.H., Wu L. (2012). Persistent sodium current and Na^+^/H^+^ exchange contributes to the augmentation of the reverse Na^+^/Ca^2+^ exchange during hypoxia or acute ischemia in ventricular myocytes. Pflug. Arch.-Eur. J. Physiol..

[204] Shimoda L.A., Polak J. (2011). Hypoxia. 4. Hypoxia and ion channel function. Am. J. Physiol.-Cell Physiol..

[205] Hammarstrom A.K., Gage P.W. (2002). Hypoxia and persistent sodium current. Eur. Biophys. J..

[206] Carmeliet E. (1999). Cardiac Ionic Currents and Acute Ischemia: From Channels to Arrhythmias. Physiol. Rev..

[207] Wang W., Ma J., Zhang P., Luo A. (2007). Redox reaction modulates transient and persistent sodium current during hypoxia in guinea pig ventricular myocytes. Pflugers Arch..

[208] Plant L.D., Xiong D., Romero J., Dai H., Goldstein S.A. (2020). Hypoxia Produces Pro-arrhythmic Late Sodium Current in Cardiac Myocytes by SUMOylation of NaV1.5 Channels. Cell Rep..

[209] Ward C.A., Giles W.R. (1997). Ionic mechanism of the effects of hydrogen peroxide in rat ventricular myocytes. J. Physiol..

[210] Song Y., Shryock J.C., Wu L., Belardinelli L. (2004). Antagonism by Ranolazine of the Pro-Arrhythmic Effects of Increasing Late INa in Guinea Pig Ventricular Myocytes. J. Cardiovasc. Pharmacol..

[211] Song Y., Shryock J.C., Wagner S., Maier L.S., Belardinelli L. (2006). Blocking Late Sodium Current Reduces Hydrogen Peroxide-Induced Arrhythmogenic Activity and Contractile Dysfunction. J. Pharmacol. Exp. Ther..

[212] Pignier C., Revenaz C., Rauly-Lestienne I., Cussac D., Delhon A., Gardette J., Le Grand B. (2007). Direct protective effects of poly-unsaturated fatty acids, DHA and EPA, against activation of cardiac late sodium current. Basic Res. Cardiol..

[213] Burnashev N.A., Undrovinas A.I., Fleidervish I.A., Makielski J.C., Rosenshtraukh L.V. (1991). Modulation of cardiac sodium-channel gating by lysophosphatidylcholine. J. Mol. Cell. Cardiol..

[214] Ahern G.P., Hsu S.-F., Klyachko V.A., Jackson M.B. (2000). Induction of Persistent Sodium Current by Exogenous and Endogenous Nitric Oxide. J. Biol. Chem..

[215] Cheng J., Valdivia C.R., Vaidyanathan R., Balijepalli R.C., Ackerman M.J., Makielski J.C. (2013). Caveolin-3 suppresses late sodium current by inhibiting nNOS-dependent S-nitrosylation of SCN5A. J. Mol. Cell. Cardiol..

[216] Bussey C.T., Erickson J.R. (2018). Physiology and pathology of cardiac CaMKII. Curr. Opin. Physiol..

[217] Matasic D.S., Yoon J.Y., McLendon J.M., Mehdi H., Schmidt M.S., Greiner A.M., Quinones P., Morgan G.M., Boudreau R.L., Irani K. (2020). Modulation of the cardiac sodium channel Na(V)1.5 peak and late currents by NAD(+) precursors. J. Mol. Cell. Cardiol..

[218] Morris C.E., Juranka P.F. (2007). Nav Channel Mechanosensitivity: Activation and Inactivation Accelerate Reversibly with Stretch. Biophys. J..

[219] Beyder A., Strege P.R., Reyes S., Bernard C.E., Terzic A., Makielski J.C., Ackerman M.J., Farrugia G. (2012). Ranolazine Decreases Mechanosensitivity of the Voltage-Gated Sodium Ion Channel Na V 1.5. Circulation.

[220] Strege P., Beyder A., Bernard C., Crespo-Diaz R., Behfar A., Terzic A., Ackerman M., Farrugia G. (2012). Ranolazine inhibits shear sensitivity of endogenous Na^+^current and spontaneous action potentials in HL-1 cells. Channels.

[221] Nassal D., Yu J., Min D., Lane C., Shaheen R., Gratz D., Hund T. (2021). Regulation of Cardiac Conduction and Arrhythmias by Ankyrin/Spectrin-Based Macromolecular Complexes. J. Cardiovasc. Dev. Dis..

[222] Dalton G.R., Jones J.V., Evans S.J., Levi A.J. (1997). Wall stress-induced arrhythmias in the working rat heart as left ventricular hypertrophy regresses during captopril treatment. Cardiovasc. Res..

[223] Salmon A.H., Mays J.L., Dalton G.R., Jones J.V., Levi A.J. (1997). Effect of streptomycin on wall-stress-induced arrhythmias in the working rat heart. Cardiovasc. Res..

[224] Parker K.K., Lavelle J.A., Taylor L.K., Wang Z., Hansen D.E. (2004). Stretch-induced ventricular arrhythmias during acute ischemia and reperfusion. J. Appl. Physiol..

[225] Cortada E., Serradesanferm R., Brugada R., Verges M. (2021). The voltage-gated sodium channel beta 2 subunit associates with lipid rafts by S-palmitoylation. J. Cell Sci..

[226] Namadurai S., Yereddi N.R., Cusdin F.S., Huang C.L.-H., Chirgadze D.Y., Jackson A.P. (2015). A new look at sodium channel β subunits. Open Biol..

[227] Nevin S.T., Lawrence N., Nicke A., Lewis R.J., Adams D.J. (2021). Functional modulation of the human voltage-gated sodium channel Na(V)1.8 by auxiliary beta subunits. Channels.

[228] Abramochkin D.V., Filatova T.S., Pustovit K.B., Dzhumaniiazova I., Karpushev A.V. (2020). Small G—protein RhoA is a potential inhibitor of cardiac fast sodium current. J. Physiol. Biochem..

[229] Petitprez S., Zmoos A.F., Ogrodnik J., Balse E., Raad N., El-Haou S., Albesa M., Bittihn P., Luther S., Lehnart S.E. (2011). SAP97 and Dystrophin Macromolecular Complexes Determine Two Pools of Cardiac Sodium Channels Na(v)1.5 in Cardiomyocytes. Circ. Res..

[230] Tang B., Hu Y., Wang Z., Cheng C., Wang P., Liang L., Xiong H., Luo C., Xu C., Chen Q. (2019). UBC9 regulates cardiac sodium channel Na(v)1.5 ubiquitination, degradation and sodium current density. J. Mol. Cell. Cardiol..

[231] Chen L., He Y., Wang X., Ge J., Li H. (2021). Ventricular voltage-gated ion channels: Detection, characteristics, mechanisms, and drug safety evaluation. Clin. Transl. Med..

[232] Remme C.A. (2013). Cardiac sodium channelopathy associated with SCN5A mutations: Electrophysiological, molecular and genetic aspects. J. Physiol.-Lond..

[233] Lowe J.S., Stroud D.M., Yang T., Hall L., Atack T.C., Roden D.M. (2012). Increased late sodium current contributes to long QT-related arrhythmia susceptibility in female mice. Cardiovasc. Res..

[234] Bezzina C.R., Remme C.A. (2008). Dilated Cardiomyopathy due to Sodium Channel Dysfunction What Is the Connection?. Circ. -Arrhythmia Electrophysiol..

[235] Huang B., El-Sherif T., Gidh-Jain M., Qin D., El-Sherif N. (2001). Alterations of Sodium Channel Kinetics and Gene Expression in the Postinfarction Remodeled Myocardium. J. Cardiovasc. Electrophysiol..

[236] Ling H., Zhang T., Pereira L., Means C.K., Cheng H., Gu Y., Dalton N.D., Peterson K.L., Chen J., Bers D. (2009). Requirement for Ca^2+^/calmodulin-dependent kinase II in the transition from pressure overload-induced cardiac hypertrophy to heart failure in mice. J. Clin. Investig..

[237] Backs J., Backs T., Neef S., Kreusser M.M., Lehmann L.H., Patrick D.M., Grueter C.E., Qi X., Richardson J.A., Hill J.A. (2009). The delta isoform of CaM kinase II is required for pathological cardiac hypertrophy and remodeling after pressure overload. Proc. Natl. Acad. Sci. USA.

[238] Undrovinas A.I., Maltsev V.A., Sabbah H.N. (1999). Repolarization abnormalities in cardiomyocytes of dogs with chronic heart failure: Role of sustained inward current. Cell. Mol. Life Sci..

[239] January C.T., Riddle J.M. (1989). Early afterdepolarizations: Mechanism of induction and block. A role for L-type Ca^2+^ current. Circ. Res..

[240] Marban E., Robinson S.W., Wier W.G. (1986). Mechanisms of arrhythmogenic delayed and early afterdepolarizations in ferret ventricular muscle. J. Clin. Investig..

[241] Szabo B., Sweidan R., Rajagopalan C.V., Lazzara R. (1994). Role of Na^+^: Ca^2+^Exchange Current in Cs+-Induced Early Afterdepolarizations in Purkinje Fibers. J. Cardiovasc. Electrophysiol..

[242] Szabo B., Kovacs T., Lazzara R. (1995). Role of Calcium Loading in Early Afterdepolarizations Generated by Cs+in Canine and Guinea Pig Purkinje Fibers. J. Cardiovasc. Electrophysiol..

[243] Furukawa T., Kimura S., Furukawa N., Bassett A.L., Myerburg R.J. (1992). Potassium rectifier currents differ in myocytes of endocardial and epicardial origin. Circ. Res..

[244] Liu D.W., Antzelevitch C. (1995). Characteristics of the delayed rectifier current (I-Kr and I-Ks) in canine ventricular epicardial, midmyocardial, and endocardial myocytes—A weaker I-Ks contributes to the longer action-potential of the M-cell. Circ. Res..

[245] Wettwer E., Amos G.J., Posival H., Ravens U. (1994). Transient outward current in human ventricular myocytes of supepicardial and subendocardial origin. Circ. Res..

[246] Kass R.S., Lederer W.J., Tsien R.W., Weingart R. (1978). Role of calcium-ions in transient inward currents and after contractions induced by strophantidin in cardiac Purkinje-fibers. J. Physiol. -Lond..

[247] Zygmunt A.C., Goodrow R.J., Weigel C.M. (1998). I NaCa andI Cl(Ca)contribute to isoproterenol-induced delayed afterdepolarizations in midmyocardial cells. Am. J. Physiol. Circ. Physiol..

[248] Volders P.G., Kulcsár A., Vos M.A., Sipido K.R., Wellens H.J., Lazzara R., Szabo B. (1997). Similarities between early and delayed afterdepolarizations induced by isoproterenol in canine ventricular myocytes. Cardiovasc. Res..

[249] Priori S.G., Corr P.B. (1990). Mechanisms underlying early and delayed afterdepolarizations induced by catecholamines. Am. J. Physiol. Circ. Physiol..

[250] January C.T., Fozzard H.A. (1988). Delayed afterdepolarizations in heart muscle: Mechanisms and relevance. Pharmacol. Rev..

[251] Bányász T., Fülöp L., Magyar J., Szentandrássy N., Varró A., Nánási P.P. (2003). Endocardial versus epicardial differences in L-type calcium current in canine ventricular myocytes studied by action potential voltage clamp. Cardiovasc. Res..

[252] Nattel S., Dobrev D. (2012). The multidimensional role of calcium in atrial fibrillation pathophysiology: Mechanistic insights and therapeutic opportunities. Eur. Heart J..

[253] Van Wagoner D.R., Pond A., Lamorgese M., Rossie S.S., McCarthy P.M., Nerbonne J.M. (1999). Atrial L-Type Ca^2+^ Currents and Human Atrial Fibrillation. Circ. Res..

[254] Biliczki P., Virág L., Iost N., Papp J.G., Varró A. (2002). Interaction of different potassium channels in cardiac repolarization in dog ventricular preparations: Role of repolarization reserve. J. Cereb. Blood Flow Metab..

[255] Litovsky S.H., Antzelevitch C. (1988). Transient outward current prominent in canine ventricular epicardium but not endocardium. Circ. Res..

[256] Liu D.W., Gintant G.A., Antzelevitch C. (1993). Ionic bases for electrophysiological distinctions among epicardial, mid-myocardial, and endocardial myocytes from the free wall of the canine left-ventricle. Circ. Res..

[257] Szentandrássy N., Banyasz T., Biro T., Szabo G., Tóth B.I., Magyar J., Lazar J., Varro A., Kovacs L., Nánási P.P. (2005). Apico?basal inhomogeneity in distribution of ion channels in canine and human ventricular myocardium. Cardiovasc. Res..

[258] Bauer A., Becker R., Karle C., Schreiner K.D., Senges J.C., Voss F., Kraft P., Kuebler W., Schoels W. (2002). Effects of the I-Kr-blocking agent dofetilide and of the I-Ks-blocking agent chromanol 293b on regional disparity of left ventricular repolarization in the intact canine heart. J. Cardiovasc. Pharmacol..

[259] Cheng J.H., Kamiya K., Liu W.R., Tsuji Y., Toyama J., Kodama I. (1999). Heterogeneous distribution of the two components of delayed rectifier K+ current: A potential mechanism of the proarrhythmic effects of methanesulfonanilide class III agents. Cardiovasc. Res..

[260] Kannel W., Wolf P., Benjamin E., Levy D. (1998). Prevalence, incidence, prognosis, and predisposing conditions for atrial fibrillation: Population-based estimates. Am. J. Cardiol..

[261] Benjamin E.J., Wolf P.A., D’Agostino R.B., Silbershatz H., Kannel W.B., Levy D. (1998). Impact of Atrial Fibrillation on the Risk of Death: The Framingham Heart Study. Circulation.

[262] Blok M., Boukens B.J. (2020). Mechanisms of Arrhythmias in the Brugada Syndrome. Int. J. Mol. Sci..

[263] Hu D., Barajas-Martínez H., Pfeiffer R., Dezi F., Pfeiffer J., Buch T., Betzenhauser M.J., Belardinelli L., Kahlig K.M., Rajamani S. (2014). Mutations in SCN10A Are Responsible for a Large Fraction of Cases of Brugada Syndrome. J. Am. Coll. Cardiol..

[264] Cano J., Zorio E., Mazzanti A., Arnau M., Trenor B., Priori S.G., Saiz J., Romero L. (2020). Ranolazine as an Alternative Therapy to Flecainide for SCN5A V411M Long QT Syndrome Type 3 Patients. Front. Pharmacol..

[265] Barake W., Giudicessi J.R., Asirvatham S.J., Ackerman M.J. (2020). Purkinje system hyperexcitability and ventricular arrhythmia risk in type 3 long QT syndrome. Heart Rhythm.

[266] Giudicessi J.R., Wilde A.A., Ackerman M.J. (2018). The genetic architecture of long QT syndrome: A critical reappraisal. Trends Cardiovasc. Med..

[267] McNair W.P., Ku L., Taylor M.R.G., Fain P.R., Dao D., Wolfel E., Mestroni L., Familial Cardiomyopathy Registry Research Group (2004). SCN5A mutation associated with dilated cardiomyopathy, conduction disorder, and arrhythmia. Circulation.

[268] Olson T.M., Michels V.V., Ballew J.D., Reyna S.P., Karst M.L., Herron K.J., Horton S.C., Rodeheffer R.J., Anderson J.L. (2005). Sodium channel mutations and susceptibility to heart failure and atrial fibrillation. J. Am. Med. Assoc..

[269] Groenewegen W.A., Wilde A.A.M. (2005). Letter regarding article by McNair et al., “SCN5A mutation associated with dilated cardiomyopathy, conduction disorder, and arrhythmia”. Circulation.

[270] Frustaci A., Priori S.G., Pieroni M., Chimenti C., Napolitano C., Rivolta I., Sanna T., Bellocci F., Russo M.A. (2005). Cardiac Histological Substrate in Patients with Clinical Phenotype of Brugada Syndrome. Circulation.

[271] Ge J., Sun A., Paajanen V., Wang S., Su C., Yang Z., Li Y., Wang S., Jia J., Wang K. (2008). Molecular and Clinical Characterization of a Novel SCN5A Mutation Associated with Atrioventricular Block and Dilated Cardiomyopathy. Circ. Arrhythmia Electrophysiol..

[272] Gosselin-Badaroudine P., Keller D.I., Huang H., Pouliot V., Chatelier A., Osswald S., Brink M., Chahine M. (2012). A Proton Leak Current through the Cardiac Sodium Channel Is Linked to Mixed Arrhythmia and the Dilated Cardiomyopathy Phenotype. PLoS ONE.

[273] Sedaghat-Hamedani F., Rebs S., El-Battrawy I., Chasan S., Krause T., Haas J., Zhong R., Liao Z., Xu Q., Zhou X. (2021). Identification of *SCN5a* p.C335R Variant in a Large Family with Dilated Cardiomyopathy and Conduction Disease. Int. J. Mol. Sci..

[274] Asatryan B. (2019). Cardiac Sodium Channel Dysfunction and Dilated Cardiomyopathy: A Contemporary Reappraisal of Pathophysiological Concepts. J. Clin. Med..

[275] Calloe K., Broendberg A.K., Christensen A.H., Pedersen L.N., Olesen M.S., Tejada M.D., Friis S., Thomsen M.B., Bundgaard H., Jensen H.K. (2018). Multifocal atrial and ventricular premature contractions with an increased risk of dilated cardiomyopathy caused by a Na(v)1.5 gain-of-function mutation (G213D). Int. J. Cardiol..

[276] Zaklyazminskaya E., Dzemeshkevich S. (2016). The role of mutations in the SCN5A gene in cardiomyopathies. Biochim. Et Biophys. Acta-Mol. Cell Res..

[277] Moreau A., Gosselin-Badaroudine P., Boutjdir M., Chahine M. (2015). Mutations in the Voltage Sensors of Domains I and II of Nav1.5 that are Associated with Arrhythmias and Dilated Cardiomyopathy Generate Gating Pore Currents. Front. Pharmacol..

[278] Zhang P.-P., Guo Z.-F., Liu Z.-P., Song L., Zhang Z.-F., Jia Y.-Z., Cao Z.-Z., Ma J.-H. (2020). Eleutheroside B, a selective late sodium current inhibitor, suppresses atrial fibrillation induced by sea anemone toxin II in rabbit hearts. Acta Pharmacol. Sin..

[279] Munger M.A., Mandroli J., Kim K., Biskupiak J., Veeraraghavan R., Gyorke S., Radwanski P. (2019). Neuronal Na^+^ Channel Inhibitor Riluzole Prevents Atrial Fibrillation in Humans. Circulation.

[280] Brand S., Seeger T., Alzheimer C. (2000). Enhancement of persistent Na^+^current by sea anemone toxin (ATX II) exerts dual action on hippocampal excitability. Eur. J. Neurosci..

[281] Chahine M., Plante E., Kallen R. (1996). Sea Anemone Toxin (ATX II) Modulation of Heart and Skeletal Muscle Sodium Channel α-Subunits Expressed in tsA201 Cells. J. Membr. Biol..

[282] Luo A., Ma J., Song Y., Qian C., Wu Y., Zhang P., Wang L., Fu C., Cao Z., Shryock J.C. (2014). Larger late sodium current density as well as greater sensitivities to ATX II and ranolazine in rabbit left atrial than left ventricular myocytes. Am. J. Physiol. Circ. Physiol..

[283] Oliveira J.S., Redaelli E., Zaharenko A.J., Cassulini R.R., Konno K., Pimenta D.C., Freitas J.C., Clare J.J., Wanke E. (2004). Binding specificity of sea anemone toxins to Na(v)1.1-1.6 sodium channels—Unexpected contributions from differences in the IV/S3-S4 outer loop. J. Biol. Chem..

[284] Klinger A.B., Eberhardt M., Link A.S., Namer B., Kutsche L.K., Schuy E.T., Sittl R., Hoffmann T., Alzheimer C., Huth T. (2012). Sea-Anemone Toxin ATX-II Elicits A-Fiber-Dependent Pain and Enhances Resurgent and Persistent Sodium Currents in Large Sensory Neurons. Mol. Pain.

